# Phytochemical Profile and Bioactive Potential of *Erythroxylum nummularium* Peyr: Evaluation of Antioxidant, Antimicrobial, and Antinociceptive Properties

**DOI:** 10.3390/antiox15081027

**Published:** 2026-08-18

**Authors:** Nilma Santos da Silva, Ícaro Tuyá Caires Amorim, Flávio Mendes de Souza, Brenda Oliveira Lima, Talita Costa dos Santos, Djalma Menezes de Oliveira, Lucas Miranda Marques, Mariluze Peixoto Cruz, Anaildes Lago de Carvalho, Regiane Yatsuda, Bruno Oliveira Moreira

**Affiliations:** 1 Multidisciplinary Institute in Health, Federal University of Bahia, Center for Natural Sciences and Biodiversity, Vitória da Conquista 45029-094, BA, Brazillucasm@ufba.br (L.M.M.); mariluze@ufba.br (M.P.C.); yatsuda@ufba.br (R.Y.); 2 Department of Science and Technology, State University of Southwest Bahia, Jequié 45208-091, BA, Brazil; 3 Department of Natural Sciences, State University of Southwest Bahia, Vitória da Conquista 45031-300, BA, Brazil; anaildes.carvalho@uesb.edu.br

**Keywords:** GC–MS profiling, antinociceptive activity, acute toxicity, *Artemia salina*, correlation analysis, total phenolic content, total flavonoid content, alkaloids

## Abstract

*Erythroxylum nummularium* is a native Brazilian species with limited phytochemical and pharmacological characterization. This study aimed to investigate the chemical composition and biological properties of methanolic extracts and fractions obtained from the leaves, branches, stem bark, and stem of the plant. Total phenolic content (TPC), total flavonoid content (TFC), total alkaloid content (TAT), and GC–MS profiling were combined with antioxidant (DPPH, β-carotene bleaching, TAC), antimicrobial, toxicological, and antinociceptive assays. The extracts exhibited substantial variation in TPC, TFC, and metabolite profiles, which strongly influenced their biological activities. The ethyl acetate fraction from the leaves (ELEN) showed the highest TPC and TFC, correlating with superior antioxidant performance, antimicrobial activity against *Staphylococcus aureus*, low toxicity in *Artemia salina*, and significant antinociceptive effects in mice, with an LD_50_ above 2000 mg/kg. In the antioxidant assays, the ethyl acetate fraction from the stem bark (ESBEN) exhibited the highest overall activity, likely due to its elevated levels of highly polar phenolics and flavonoids. Among the fractions characterized by GC–MS, DSBEN, DLEN, and DBEN were the most active, with phenolic acids, their methylated derivatives, and low-molecular-weight organic acids contributing synergistically to antioxidant activity. These findings identify *E. nummularium* as a promising source of structurally diverse metabolites with antioxidant, antimicrobial, and antinociceptive potential and support further investigation toward the development of natural bioactive agents.

## 1. Introduction

The prevalence of metabolic diseases worldwide has been a longstanding issue for centuries. These physiological disorders are characterized by changes in mitochondria, organelles that produce ATP in cells [1]. Such conditions lead to reduced oxidative capacity and impaired detoxification of reactive oxygen species (ROS) [2].

Exacerbated ROS production can lead to pathophysiological states resulting from oxidative stress. The central dysfunctions associated with this phenomenon include high blood pressure, hyperglycemia, abdominal obesity, dyslipidemia, Parkinson’s disease, Alzheimer’s disease, cancer, and inflammation [1,3,4,5,6,7].

Given the significant increase in the number of affected patients and the rising costs of healthcare, particularly amid growing pressure on the public healthcare system, identifying new bioactives with minimal side effects is increasingly essential [8]. In this context, recent studies have highlighted the efficacy of species of the genus *Erythroxylum* [9,10,11,12].

Among the various species with therapeutic properties, *Erythroxylum nummularium* Peyr stands out. This plant belongs to a widely recognized genus for its medicinal properties. The popularity of the genus *Erythroxylum* is due, in large part, to the millennial use of the leaves of *Erythroxylum coca* Lam by the natives of South America. In this context, they used this plant as a stimulant and source of well-being [11]. The genus *Erythroxylum*, the most prominent within the Erythroxylaceae family, is notable for its diverse array of bioactive compounds with pharmacological relevance [12]. The main chemical compounds in this genus include flavonoids, diterpenes, alkaloids, norisoprenoids, phenolic derivatives, steroids, triterpenoids, and tannins [12,13]. This genus’s rich diversity of secondary metabolites includes compounds with broad applicability across agriculture, industrial processes, nutritional products [11], and pharmacology [12], underscoring its multidisciplinary relevance.

Phytochemical investigations of various *Erythroxylum* species have consistently demonstrated their broad pharmacological potential. For instance, *E. vacciniifolium* Mart. is known for its tonic and aphrodisiac effects [14], with additional benefits in the treatment of erectile dysfunction and antiviral activity against Human Immunodeficiency Virus (HIV). Moreover, this species exhibits antimicrobial and cytotoxic properties, further reinforcing its therapeutic relevance [11,14]. Several other species within the genus display multifaceted biological activities. *E. ovalifolium* Peyr., for example, has been shown to neutralize snake venom, reduce edema, control hemorrhages, and exert antifungal effects [10,11]. Similarly, *E. suberosum* A. St. Hill and *E. laurifolium* Lam. contain compounds with anesthetic, antioxidant, cytotoxic, antidiabetic, antihypertensive, antiviral, and antimicrobial activities [11,15,16,17]. The pharmacological profiles of *E. pervillei* Baill., *E. macrocarpum* O. E. Schulz, and *E. caatingae* Plowman have also been explored. Studies have highlighted their efficacy in managing conditions such as diabetes, neoplasms, tumors, and abdominal pain [11,18,19]. Additionally, antimicrobial, antifungal, and antibiotic activities have been reported for *E. macrocarpum* and *E. caatingae* [18,20], further expanding the therapeutic potential of the genus.

Expanding phytochemical studies of the genus, some research has begun to elucidate the chemical composition of *E. nummularium*. Barreiros et al. identified in the leaves, so far the only plant organ investigated, the compounds quercetin, 4′,7-dimethoxy-3′,5-dihydroxyflavanol (also known as 7,4′-dimethylquercetin), and epicatechin, whose antioxidant activities were evaluated [21]. These authors also reported the presence of quercetin-3-O-D-glucopyranoside and 14-methoxyryanodanol, the latter exhibiting insecticidal activity against *Aedes aegypti* larvae [22]. Additionally, β-amyrin palmitate and stearate, erythrodiol palmitate and stearate, oleanolic acid palmitate, and β-sitosterol were isolated [23]. These findings reinforce the phytochemical richness of *E. nummularium* and highlight the need for broader investigations encompassing other plant organs and biological activities.

Polyphenols are secondary metabolites widely recognized for their diverse and well-established biological activities [24]. Their antioxidant, antibacterial, anti-inflammatory, and anticancer properties have garnered significant scientific attention in recent years [25]. Among them, gallic acid, a phenolic compound identified in *E. nummularium* leaves, has demonstrated potent antioxidant and anti-inflammatory effects, including the ability to neutralize free radicals, reduce oxidative stress, and modulate inflammatory pathways, reinforcing its therapeutic relevance in oxidative stress-related disorders [26].

In the context of oxidative stress, polyphenols have been recognized for their ability to neutralize free radicals, which play a central role in the development of certain pathophysiological conditions [27]. The elevation of antioxidant enzyme activity and the suppression of ROS production are essential mechanisms by which polyphenols exert their antioxidant effect, contributing to the prevention of cell damage. In addition, evidence suggests that flavonoids, a primary class of polyphenols, may be effective in preventing and treating neuroinflammatory disorders, while also reducing apoptosis and combating motor impairments [28]. Therefore, the recognition of new classes of flavonoids in plant species may be essential for suppressing other diseases and stimulating preclinical trials. Thus, the discovery of new classes of flavonoids in plant species may represent a breakthrough in the development of innovative therapies for various health conditions.

Thus, even with the Ethnopharmacological importance of the genus *Erythroxylum* already established, there are no studies on the medicinal potential of *E. nummularium*. Therefore, the present study proposes to investigate the chemical composition and biological activities of the leaves, twigs, stem bark, and stem of this species. The methanolic extracts and organic phases were submitted to preliminary phytochemical analyses. Chromatographic techniques, such as thin-layer and gas chromatography coupled with mass spectrometry, were employed. In addition, antioxidant capacity assays, quantification of phenolics and total flavonoids, evaluation of toxicity against *Artemia salina* Leach, and investigation of antimicrobial and antinociceptive activities in animal models were performed. This approach aims to fill the knowledge gap about this plant species and provide insights into its therapeutic potential, thereby contributing to the development of new drugs based on natural products.

## 2. Materials and Methods

### 2.1. Materials and Reagents

The GC/MS-QP2010SE mass spectrometer (Shimadzu, Kyoto, Japan) was used for gas chromatography analyses. All weighing operations were performed using the ATY224 analytical balance (Shimadzu, Kyoto, Japan). The reading of absorbances in the experiments was conducted using a UV-vis spectrophotometer (Bel Engineering, Monza, Italy), and exposure to ultraviolet light was performed in a dark room equipped with an ultraviolet light source (Biotech, Piracicaba, Brazil). The sterilization drying oven (Vulcan, São Paulo, Brazil) was used for sterilization and drying processes, and centrifugation was performed in a 32A Centrifuge (Hettich, Tuttlingen, Germany). The samples were homogenized using a vortex shaker (Prolab Laboratory Materials, São Paulo, Brazil), and pH measurements were taken with a pH meter (MS Tecnopon, Piracicaba, Brazil). Methanol, ethanol, dichloromethane, chloroform, ethyl acetate, hexane, and other reagents were of analytical grade and purchased from ACS Scientific, Exodus, and Synth (São Paulo, Brazil). The analytical-grade reagents DPPH, Folin–Ciocalteu, β-carotene, Tween 80, and pyridine used in the tests were purchased from Merck (Frankfurter, Germany). Aluminum chloride was acquired from Neon (Rio de Janeiro, Brazil), while bromocresol green, ascorbic acid, and acetic acid were obtained from Dinâmica (Indaiatuba, Brazil). Linolenic acid was purchased from Nature Essential (Sevilha, Spain), and BHI Agar (Brain Heart Infusion Agar) was purchased from Prolab Laboratory Materials (São Paulo, Brazil). The reagents used for derivatization by silylation were pyridine (Dinâmica), N,O-Bis(trimethylsilyl) trifluoroacetamide (BSTFA), and trimethylchlorosilane (TMCS) (Merck). The extracts and organic phases were monitored by thin-layer chromatography (TLC) using silica gel 60 F_254_ (Merck), and the spots were visualized as described in Section 2.5. The *Artemia salina* eggs were donated by the State University of Southwest Bahia (UESB).

### 2.2. Collection and Taxonomic Identification of Plant Material

The botanical species was collected in the rural area of Brejo Novo (13°55′28.50″ S, 40°6′33.40″ W) in Jequié, Bahia. The collection was coordinated by the curator of the Herbarium of the State University of Southwest Bahia (HUESB). The plant species was registered in SisGen (National System for the Management of Genetic Heritage and Associated Traditional Knowledge) under the registration code A8C8EDD. A voucher specimen (14665) was deposited at the Herbarium HUESB.

### 2.3. Preparation of Extracts

After drying at room temperature, the plant material was ground into a fine powder. The crushed material (815.836 g of leaves, 2 kg of branches, 1 kg of Stem bark, and 920 g of stem) was macerated in MeOH separately for ten consecutive extractions, each lasting approximately 48 h. The filtrate obtained was gathered and concentrated under reduced pressure, generating the methanolic extracts of the leaves (119.224 g; MLEN), branches (132.029 g; MBEN), stem bark (212.408 g; MSBEN), and stem (70.812 g; MSEN). The methanolic extracts were dissolved in MeOH/H_2_O (7:3, *v*/*v*) and partitioned between hexane, dichloromethane, ethyl acetate, and butanol. They were giving rise to the organic phases of each organ (Table 1). Both the methanolic extracts and the fractions were subjected to various tests.

### 2.4. Qualitative Phytochemical Screening

The phytochemical prospecting tests of the organic phases obtained from the methanol extract of the leaves, branches, stem bark, and stem of *E. nummularium* were carried out according to the methodologies described below. These tests were based on colorimetric visualization and/or precipitate formation upon addition of specific reagents to identify the following classes of secondary metabolites: alkaloids, flavonoids, phenolics, steroids, saponins, and tannins. The qualitative results were expressed as the presence (+) or absence (−) of the phytochemical classes.

### 2.5. Thin-Layer Chromatography Analysis (TLC)

Alkaloids: The organic phases were dissolved in dichloromethane, separately applied to the silica gel plate, and eluted with an appropriate solvent mixture. After the elution of the chromatographic plate, for the observation of alkaloids, the Wagner reagent (1 g of iodine and 10 g of potassium iodide in 100 mL of distilled water) was sprayed on the silica gel plate. The appearance of reddish-brown spots on the plaque indicated the presence of alkaloids [29].

Flavonoids and Phenolics: The presence of phenolic compounds was assessed using ferric chloride (5% in ethanol) as a chromogenic reagent. After elution of the chromatographic plate, the reagent was sprayed onto the TLC surface. The appearance of blue or dark green coloration indicated the presence of phenolic substances [29]. For flavonoid detection, 1 mL of the extract or organic fraction was treated with a few drops of sodium hydroxide solution (10% *w*/*v*). The absence of yellow coloration suggested the lack of flavonoids, whereas the formation of an intense yellow color that turned colorless upon the addition of diluted sulfuric acid confirmed their presence [29].

Steroids and terpenoids were assessed by applying the Liebermann–Burchard reagent (a mixture of 5 mL acetic anhydride and 5 mL sulfuric acid dissolved in 50 mL ethanol) to the TLC plate. After reagent application, the plate was heated gently on a hotplate, and the appearance of colored spots indicated the presence of steroids and/or terpenoids [30].

Saponins: The colorimetric method with sulfuric acid was employed to detect saponins, as described [31]. In this method, 5 mL of H_2_SO_4_ was added to 50 mL of ethanol, and the final volume was adjusted to 100 mL. After eluting the chromatographic plate, saponins were detected by spraying the reagent on the TLC plate. The presence of saponins was indicated by the formation of a purple eggplant coloration after heating.

Tannins: The reagent used was a 2% (*w*/*v*) methanolic solution of FeCl_3_ [31]. The formation of the blue or green spots on the TLC plate indicated the presence of tannins.

### 2.6. Determination of Alkaloids by the Precipitation Method

About 100 mg of each organic phase was dissolved in 5 mL of ethyl alcohol, and 2 mL of Wagner’s reagent was added. The formation of a reddish-brown precipitate indicated the presence of alkaloids [32].

### 2.7. Determination of Total Alkaloid Content (TAT)

To quantify TAT in the organic phases, 10 mg of the samples were weighed and diluted in 1 mL of HCl 2 mol L^−1^, followed by filtration as described by [33]. The filtrate was transferred to a separation funnel and washed with 10 mL of chloroform (3 times). The pH of the aqueous phase was adjusted to neutral with 0.1 mol L^−1^ NaOH. Then, 5 mL of phosphate-buffered solution (pH 4.7) and 5 mL of bromocresol green solution (0.025 mol L^−1^) were added. The mixture was stirred, and the complex formed was extracted with 2.5 mL of chloroform under vigorous stirring. The chloroform phase was collected in a 10 mL volumetric flask and supplemented with chloroform to the final volume. The absorbance of this complex was measured at 470 nm. All samples were analyzed in triplicate. A calibration curve was constructed using a 0.25 mg mL^−1^ atropine sulfate solution. Each concentration used in the construction of the curve was treated in the same manner as the samples. The results were expressed as mg of atropine equivalent per gram of the extract (mg EA g^−1^). This assay was performed only in the organic phases, presenting a positive result in the qualitative alkaloid test.

### 2.8. Total Flavonoid Content (TFC)

TFC was determined via a colorimetric assay based on the aluminum chloride method [34], with minor modifications. Initially, 4 mg of each extract or organic phase was dissolved in methanol to yield solutions with concentrations ranging from 0.4 to 0.6 mg·mL^−1^. Subsequently, 1 mL of each sample was transferred into individual Falcon tubes containing 4 mL of distilled water, followed by the addition of 300 µL of 5% *w*/*v* sodium nitrite (NaNO_2_). The mixtures were vortexed for 30 s. Next, 300 µL of 10% *w*/*v* aluminum chloride (AlCl_3_) was added, and the samples were vortexed vigorously for an additional 30 s. Afterward, 2 mL of sodium hydroxide (NaOH, 1 mol·L^−1^) was added, and the samples were allowed to stand for 15 min at room temperature before absorbance measurement. Absorbance readings were taken at 510 nm using a UV-Vis spectrophotometer.

A calibration curve was prepared using quercetin at 200, 400, 600, 800, and 1000 μg mL^−1^, following the same methodology used for the samples. All analyses were performed in triplicate. The TFC was calculated from the calibration curve using quercetin as a standard. The results were expressed in milligrams of quercetin equivalent per gram of extract (mg EQ⋅g^−1^).

### 2.9. Total Phenolic Content (TPC)

The TPC in the MeOH extracts and organic phases was quantified by the Folin–Ciocalteu method, as described by [35,36] with adaptations. An aliquot of the extracts/phases was dissolved in 80% ethanol and transferred to a 10 mL volumetric flask to provide concentration solutions between 150 and 250 μg mL^−1^. The reaction medium was prepared using 0.1 mL of the extracts/phases solution, 0.5 mL of Folin–Ciocalteu reagent (10%, *v*/*v*), and 2.0 mL of sodium carbonate (15%, *w*/*v*). Subsequently, this mixture was diluted to the final volume of 10 mL with distilled water. After 1 h of reaction, protected from light, the absorbances of the samples were measured at 760 nm, with ethanol and all reagents except the extract/phase as blank. A calibration curve was prepared using gallic acid at concentrations of 50, 75, 100, 125, and 150 μg mL^−1^, following the same methodology as the samples. All analyses were performed in triplicate. The TPC concentration was calculated from a calibration curve using pure gallic acid as the standard. Results were expressed as milligram gallic acid equivalents per gram of extract (mg EGA⋅g^−1^).

### 2.10. DPPH Radical Scavenging Assay

The crude extracts and organic phases of *E. nummularium* were submitted to the DPPH (2,2-diphenyl-1-picrylhydrazyl) free radical scavenging test [37], with adaptations. The methanolic extracts were prepared at concentrations of 50, 100, 150, 200, and 250 µg mL^−1^, while the DPPH solution was prepared at a concentration of 40 µg mL^−1^. For each concentration of the extracts, a reaction medium was prepared in triplicate, containing 200 µL of crude extract or organic phases, or methanol for the control, along with 1800 µL of DPPH solution. The samples were incubated at room temperature, protected from light, for 30 min. Next, absorbances were measured at 515 nm in a spectrophotometer. The antioxidant capacity of the extracts was evaluated by the DPPH method, following the classification of [38], which considers EC_50_ in three categories: good activity when the EC_50_ value of the extract is up to three times higher than the standard used, medium activity if it between three and seven times above the EC_50_ of the standard, and low activity if the EC_50_ is seven times higher than the standard used.

### 2.11. β-Carotene/Linolenic Acid System

The antioxidant activity assay using the β-carotene/linolenic acid system was performed according to the method described by Moreira et al. [37], with some adaptations. Initially, 2.6 mg of β-carotene was dissolved in 2 mL of chloroform. This solution was transferred to a volumetric flask containing 403 mg of Tween 80 emulsifier and 42.8 mg of linolenic acid. The chloroform was removed by rotary evaporation, and then 200 mL of distilled water was added, with vigorous stirring for 2 min, to form an emulsion, which constitutes the oxidizing medium. An aliquot of 2.7 mL of the oxidizing medium was added to a cuvette containing 300 µL of the samples in triplicate. The extracts and phases were prepared at concentrations of 50, 100, 150, 200, and 250 µg mL^−1^ using methanol as the solvent. Absorbances were measured at 470 nm immediately after mixing, and the samples were subsequently incubated in a water bath at 50 °C for 1 h for the second reading. The control was prepared with 2.7 mL of oxidizing medium and 300 µL of methanol. For the calibration curve, ascorbic acid was used in the same concentrations as the extracts. Antioxidant activity (AA) was calculated using the following equation:

AA = (DR_C_ − DR_A_)/DR_C_ × 100.

where DRC is the rate of degradation of the control (DR_C_ = ln (a/b)/60), and DRA is the rate of degradation in the presence of the sample (DR_A_ = ln (a/b)/60). In the equation, “a” represents the initial absorbances at time 0, and “b” is the absorbance after 60 min. The efficient concentration, the amount of antioxidant needed to reduce the percentage of oxidation inhibition by 50% (EC_50_), was calculated by linear regression, where the abscissa axis represented the sample concentration (µg mL^−1^) or the concentrations of the positive control (ascorbic acid), and the ordinate axis represented the AA.

### 2.12. Total Antioxidant Capacity (TAC)

TAC analysis with modifications was performed using the phosphomolybdate method [39,40]. The extracts, organic phases, and quercetin and gallic acid patterns were prepared at concentrations of 50, 100, 150, 200, and 250 µg/mL, using methanol as the solvent. For each concentration prepared, an aliquot of 0.25 mL was transferred to a test tube, to which 2.5 mL of the reagent solution was added. The reagent solution consisted of 0.6 M sulfuric acid, 28 mM sodium phosphate, and four mM ammonium molybdate. The test tubes were then capped and incubated in a water bath at 90 °C for 90 min. After incubation, the samples were cooled to room temperature. The absorbance of the samples was measured at 765 nm using a spectrophotometer. All analyses were performed in triplicate. Initially, the samples’ CAT percentage was calculated by comparing them to ascorbic acid at a concentration of 200 μg mL^−1^, considered 100% (control). The following equation was used to calculate the percentage of TAC:

% CAT = [(Abs_Sample_ − Abs_blank_)/(Abs_control_ − Abs_blank_)] × 100.

where Abs_sample_ is the absorbance of the sample, Abs_blank_ is the absorbance of the blank, and Abs_control_ is the absorbance of ascorbic acid 200 μg mL^−1^ (control).

The results of the TAC evaluation were expressed as efficient concentration (EC_50_), which is the amount of antioxidants needed to reduce the percentage of oxidation inhibition by 50%. EC_50_ was calculated by linear regression, where the abscissa axis represented the concentration of the samples (µg/mL) or the concentration of the standards (quercetin and gallic acid), and the ordinate axis represented the % TAC.

### 2.13. Heat Map: Correlation of Total Phenolic and Flavonoid Content with Antioxidant Assays

The heat map was generated using GraphPad 8.0.1 software (GraphPad Software Inc., San Diego, CA, USA). The correlations between TPC and TFCs and antioxidant activities (DPPH, β-carotene, and TAC) were evaluated using Pearson’s correlation coefficient (*r*). Normality was assessed using the Shapiro–Wilk test and by visual inspection of Q–Q plots. Some variables did not meet the normality assumption (Shapiro–Wilk, *p* ≤ 0.05). For these cases, Spearman’s rank correlation was used instead. *p* values lower than 0.05 were considered statistically significant.

### 2.14. Chemical Composition Analysis by GC/MS

The hexane and dichloromethane phases of the leaves, branches, stem bark, and stem of *E. nummularium* were analyzed by gas chromatography coupled with mass spectrometry (GC-MS). Before GC-MS analysis, the samples were derivatized by silylation. For derivatization, 3 mg of each organic phase was weighed, and 60 μL of pyridine was added. To this solution, 100 μL of a reactive mixture of N, O-Bis (trimethylsilyl) trifluoroacetamide (BSTFA) containing 1% trimethylchlorosilane (TMCS) was added. This mixture was heated to 70 °C for 30 min, allowing for the complete derivatization of the substances present. Then, one μL of the derivatized mix was injected into the GC-MS system. GC-MS analyses were performed using an Rtx-5MS fused silica capillary column (30 m; 0.25 mm internal diameter; 0.25 μm film) with helium as carrier gas. The injector temperature was maintained at 290 °C. The oven temperature setting started at 80 °C for 5 min, followed by an increase to 285 °C at a rate of 4 °C/min, and then remained at 285 °C for 40 min. The detector temperature was set to 200 °C, and the temperature at the GC-MS system interface was maintained at 290 °C. Mass scanning was performed over the 35–600 *m/z* range, with the ion source operating at 70 eV. The compounds were identified in the extracts by comparing the mass spectra obtained with those available in the NIST 08, FFNSC1.3, and WILEY8 databases.

### 2.15. Microorganisms and Cultivation

The microorganisms used in this study were the standard resistant strain *Staphylococcus aureus* ATCC 43300 INCQS 00306 (resistant to oxacillin) and clinical isolates of *S*. *aureus* 16A, 112, 92, and 29, collected from two public hospitals in the city of Vitória da Conquista, Bahia [41]. The strains were thawed (−80 °C) and incubated for 24 h at 37 °C in BHI broth. The strains of *Streptococcus mutans* ATCC 700610 and *Streptococcus sobrinus* were obtained from the Department of Pharmacology, Anesthesiology, and Therapeutics of the School of Dentistry, UNICAMP (University of Campinas, Piracicaba, São Paulo). The *Streptococcus mutans* ATCC 25175 strain used was kindly provided by the National Institute for Quality Control in Health (INCQS)—Oswaldo Cruz Foundation—Fiocruz (Rio de Janeiro, RJ, Brazil). These strains were thawed (−80 °C), incubated for 24 h at 37 °C and 5% of CO_2_ in BHI broth with 1% glucose. After the bacteria were developed, the minimum inhibitory concentration (MIC) and the minimum bactericidal concentration (MBC) were determined.

### 2.16. Determination of the Minimum Inhibitory Concentration (MIC)

The microdilution technique, performed in 96-well microplates, was used to determine the MIC. The inoculum of the studied strains was prepared with a final bacterial concentration of approximately 1–2 × 10^8^ CFU/mL (0.5 McFarland scale). These inoculums were diluted in heart-brain infusion broth (BHI) medium (Kasvi^©^) at a ratio of 1:1000. In the microplates, 190 μL of the inoculum-containing medium (final concentration 1–2 × 10^5^ CFU/mL) was added to each well, along with ten μL of the organic phases of all the plant organs of *E. nummularium* studied. The final concentrations of the organic phases ranged from 31.25 to 1000 μg/mL, obtained by serial dilution in a 2:1 ratio. The plates were incubated for 24 h at 37 °C and 5% of CO_2_ [41]. After incubation, visual inspection was performed to evaluate growth by assessing the medium turbidity and/or the presence of colonies at the bottom of the wells. In wells without visible growth, 20 μL of resazurin (Merck Sigma©, Rio de Janeiro, Brazil) was added. After 20 min of incubation with resazurin, the absence of microorganisms was indicated by a blue color, while a pink color indicated bacterial growth. The MIC was defined as the lowest concentration that did not show visible bacterial growth, confirmed by the blue stain after adding resazurin.

### 2.17. Determination of the Minimum Bactericidal Concentration (MBC)

MBC was determined from the wells that did not exhibit visible bacterial growth in the MIC assay for the evaluated strains. An eight μL aliquot from each well was inoculated into plates with BHI agar. Then, the plates were incubated at 37 °C and 5% CO_2_ for 24 h. After the incubation period, the plates were visually read. MBC was defined as the lowest extract concentration that caused the death of 99.9% of bacterial cells, as determined by the absence of visible bacterial growth on the agar surface [42].

### 2.18. Toxicity Test of Artemia salina Leach

A lethality assay of *A. salina* was performed to verify the toxicity of methanolic extracts and organic fractions of *E. nummularium*. The test was conducted according to the methodology described by [43], with some adaptations. The eggs of *A. salina* were incubated in artificial seawater (3.2 g/L) under continuous illumination from an incandescent lamp, maintaining a temperature of 24–26 °C. After 24 h, the cysts hatched. For the assay, ten newly hatched *A. salina* larvae were transferred to tubes containing artificial seawater and DMSO (1%), to which different concentrations of the extract (12.5–200 μg/mL) were added. Each concentration was evaluated in triplicate. After 24 h of exposure, the surviving nauplii were counted. The results were processed to calculate LD_50_ (lethal dose for 50% of the tested population) with a 95% confidence interval. All experiments were performed in triplicate.

### 2.19. Antinociceptive Activity

Male *Balb-C* mice weighing 25 and 30 g were kept in the Federal University of Bahia (UFBA) vivarium. The animals were housed in polypropylene boxes and maintained under controlled light conditions (a 12 h light/dark cycle) and an average temperature of 22 ± 3 °C. They had access to water and feed. On the day of the experiment, the mice were transferred to the laboratory 30 min before the start of the tests to allow for adaptation to the new environment. The Animal Use Ethics Committee (CEUA-IMS/CAT-UFBA) evaluated and approved this experiment under protocol 107/2022.

### 2.20. Acetic Acid-Induced Abdominal Contortions Test

The ELEN phase was evaluated in the contortions. The mice were divided into five groups, each consisting of six animals. The treatment groups received oral doses of 6.25, 12.5, 25, and 50 mg/kg of ELEN. The control group received only water. Sixty minutes after the doses were administered, the animals received an intraperitoneal injection of acetic acid (0.6%). The number of abdominal contortions, followed by torso twists and hind limb extension, was recorded over 20 min [37].

### 2.21. Acute Toxicity Studies of the ELEN Fraction

To assess the acute toxicity of the ELEN fraction, the LD_50_ method was employed after oral administration of 2000 mg/kg. Six animals were used in the experiment: three received the ELEN fraction, and three served as the control group. The animals were observed for 24 h immediately after treatment, followed by daily monitoring for 15 days to evaluate their behavior and physical condition. Observations focused on detecting symptoms such as aggression, tremors, paralysis, and skin lesions. On day 15, they were euthanized after anesthesia. Blood samples were collected for hematological analysis. Parameters evaluated included total leukocyte count, absolute and relative lymphocyte count, absolute and relative monocyte count, eosinophils, basophils, absolute neutrophils, hemoglobin concentration, hematocrit levels, total red blood cell count, platelet distribution width, and red blood cell distribution width. This study followed the methodology described by Simeonova et al. [44], with adaptations.

### 2.22. Statistical Analysis

In the tests performed with mice, the results were presented as mean ± standard deviation (S.D.) (*n* = 6 for group). Statistical comparisons between groups were initially performed using one-way analysis of variance (ANOVA), followed by the Bonferroni test for multiple comparisons, using GraphPad Prism software (version 5.0; GraphPad Software Inc., La Jolla, CA, USA). Statistical differences were considered significant if *p* < 0.05. In the toxicity experiment in mice (LD_50_), the results were expressed as mean ± SD for six animals. Complete blood count data were treated using the GraphPad Prism software, using one-way analysis of variance (ANOVA), followed by Tukey’s test. For the TAT, TFT, TPC, and antioxidant activity assays, results were expressed as the mean ± S.D. of three replicates (*n* = 3). Statistical analyses of these data followed a unidirectional analysis of variance (ANOVA) and Tukey’s test for multiple comparisons, using Statistica 13 software (Stat-Soft Inc., Tulsa, OK, USA). Results with *p* < 0.05 were considered statistically significant. In the toxicity tests with *A. salina*, LD_50_ values with 95% confidence intervals were calculated using the probit analysis method in Microcal Origin 5.0 (OriginLab Corporation, Northampton, MA, USA).

## 3. Results

### 3.1. Qualitative Phytochemical Analysis

A preliminary phytochemical investigation was conducted to identify the presence or absence of secondary metabolites in the leaf, branch, stem bark, and stem fractions. The screening revealed the presence of various substances, such as phenolics, flavonoids, saponins, and tannins. However, there are some exceptions between the fractions (Table 2). The presence of alkaloids in the fractions HLEN, DLEN, HBEN, HSBEN, DSBEN, and DSEN was revealed by precipitation analysis. However, the TLC analysis did not reveal the presence of alkaloids.

### 3.2. Determination of Total Alkaloid Content (TAT)

Table 3 presents the alkaloid content across the different fractions of *E. nummularium*. The highest concentrations were observed in the HBEN, HSBEN, and DLEN fractions, with values of 24.03 ± 0.80, 23.12 ± 0.90, and 22.67 ± 0.88 mg EA g−1, respectively.

### 3.3. Determination of Total Phenolic Content (TPC)

The quantification of total phenolic compounds in *E. nummularium* extracts was performed using the Folin–Ciocalteu method, and results are expressed as mg GAE·g^−1^. Table 4 summarizes the TPC values across different plant organs obtained from crude extracts and successive organic fractions.

The extracts and organic fractions of *E. nummularium* exhibited substantial levels of phenolic compounds, with notable variation across plant organs and solvents. The hexane fraction showed negligible phenolic content in all organs except for the leaves (13.3 ± 0.07 mg GAE·g^−1^), suggesting limited solubility of these metabolites in non-polar solvents. Similarly, low phenolic concentrations were observed in the crude stem extract (87.1 ± 3.2 mg GAE·g^−1^) and in most dichloromethane fractions, except for the stem bark (183.9 ± 6.3 mg GAE·g^−1^). In contrast, the ethyl acetate fraction yielded the highest TPC values, particularly in the stem bark (958.9 ± 23.9 mg GAE·g^−1^), followed by the leaves and branches. The butanol fraction also demonstrated considerable phenolic content across all organs, with the stem bark again showing the highest concentration (658.9 ± 21.4 mg GAE·g^−1^).

### 3.4. Determination of Total Flavonoid Content (TFC)

In the organic phases, the ethyl acetate fraction showed the best results for all organs except the stem, where the dichloromethane fraction yielded the best result. In the overall evaluation of the results, the best TFCs were obtained in the order ESBEN > MSBEN > MBEN > BSBEN.

The TFC of *E. nummularium* extracts was quantified, and the results are presented in Table 5. Among the plant organs analyzed, the stem bark exhibited the highest flavonoid concentration, whereas the stem showed the lowest. All crude extracts demonstrated elevated TFC values, except the stem extract. Regarding the organic fractions, ethyl acetate yielded the highest TFC across all organs except the stem, for which the dichloromethane fraction showed superior performance. Overall, the most prominent TFC values were observed in the following order: ESBEN > MSBEN > MBEN > BSBEN.

### 3.5. Antioxidant Activity

#### 3.5.1. DPPH Radical Scavenging Assay

The antioxidant potential of *E. nummularium* was assessed using the DPPH radical scavenging assay. Results are summarized in Table 6. The data are expressed as EC_50_ values, representing the concentration (µg·mL^−1^) required to reduce the initial DPPH radical concentration by 50%. Ascorbic acid was employed as the reference standard.

No individual organ exhibited superior antioxidant activity across all tested fractions. Nonetheless, the stem bark consistently demonstrated the greatest overall radical scavenging capacity. Among the crude extracts, all organs showed notable activity, with EC_50_ values below 100 µg·mL^−1^, except for the stem, which was inactive. The hexane fraction was inactive across all organs, indicating its inefficiency in extracting antioxidant constituents. In contrast, the ethyl acetate fraction yielded the most pronounced antioxidant effects in most organs, except the stem, where the dichloromethane fraction showed comparatively greater efficacy.

The most pronounced antioxidant responses were observed in EBEN and MSBEN, with no statistically significant difference from the standard (ascorbic acid), underscoring their strong radical scavenging potential. Subsequently, MBEN, ELEN, BSBEN, and ESBEN exhibited excellent antioxidant activity, with no statistically significant differences among them. However, their EC_50_ values were significantly higher than those of the most active fractions (EBEN and MSBEN). Following this group, MLEN also demonstrated good antioxidant potential, but its EC_50_ value differed significantly from that of the preceding group.

#### 3.5.2. β-Carotene/Linolenic Acid Method

The results of evaluating the extracts and organic phases of the *E. nummularium* organs regarding the inhibition of oxidation in the β-carotene bleaching assay are presented in Table 7. In this trial, ascorbic acid was also used as a standard. In comparing the plant organs, the leaves showed promising results, with the DLEN fraction standing out as the most effective, indicating vigorous antioxidant activity. The BLEN and ELEN fractions also showed significant activity. The branches exhibited intermediate antioxidant activity, with greater variation than the leaves. The stem showed the lowest antioxidant activity among the organs analyzed. Comparing the different fractions, the hexane fraction showed the highest EC_50_ values in all the organs tested, indicating low antioxidant activity. On the other hand, the ethyl acetate fraction showed consistently good antioxidant activity in almost all organs except the stem, standing out as one of the most promising fractions. The fractions DLEN, BLEN, ESBEN, ELEN, MBEN, BSBEN, and EBEN exhibited the best EC_50_ values, with no statistically significant differences observed. This finding suggests that all these fractions possess a comparable ability to inhibit β-carotene oxidation, demonstrating efficiency superior to the standard used in this assay.

#### 3.5.3. Total Antioxidant Capacity (TAC)

The TAC of *E. nummularium* extracts was evaluated using the phosphomolybdenum complexation method. Results are presented in Table 8, gallic acid and quercetin were used as reference standards, yielding EC_50_ values of 37.8 ± 0.2 µg·mL^−1^ and 110.3 ± 3.4 µg·mL^−1^, respectively.

Among the crude extracts, the branches (154.8 ± 7.0 µg·mL^−1^) and leaves (159.8 ± 4.3 µg·mL^−1^) exhibited the highest antioxidant activity, while the stem (462.8 ± 14.7 µg·mL^−1^) showed the lowest. Regarding the polarity of the extracting solvents, the ethyl acetate and butanol fractions showed enhanced antioxidant capacity in the stem bark (61.8 ± 2.7 µg·mL^−1^ and 126.9 ± 3.0 µg·mL^−1^, respectively). In contrast, the dichloromethane and hexane fractions yielded better results in the branches (MSBEN, 170.3 ± 8.0 µg·mL^−1^ and 250.7 ± 6.8 µg·mL^−1^, respectively).

The ethyl acetate fraction of the stem bark was the most active among all samples, with an EC_50_ value statistically equivalent to gallic acid and significantly lower than quercetin. EBEN and BSBEN also demonstrated strong antioxidant capacity, showing no significant differences from the quercetin standard. Conversely, the hexane fractions showed the weakest antioxidant activity across all organs.

### 3.6. Heat Map: Correlation of Total Phenolic and Flavonoid Content with Antioxidant Assays

**Figure 1:**
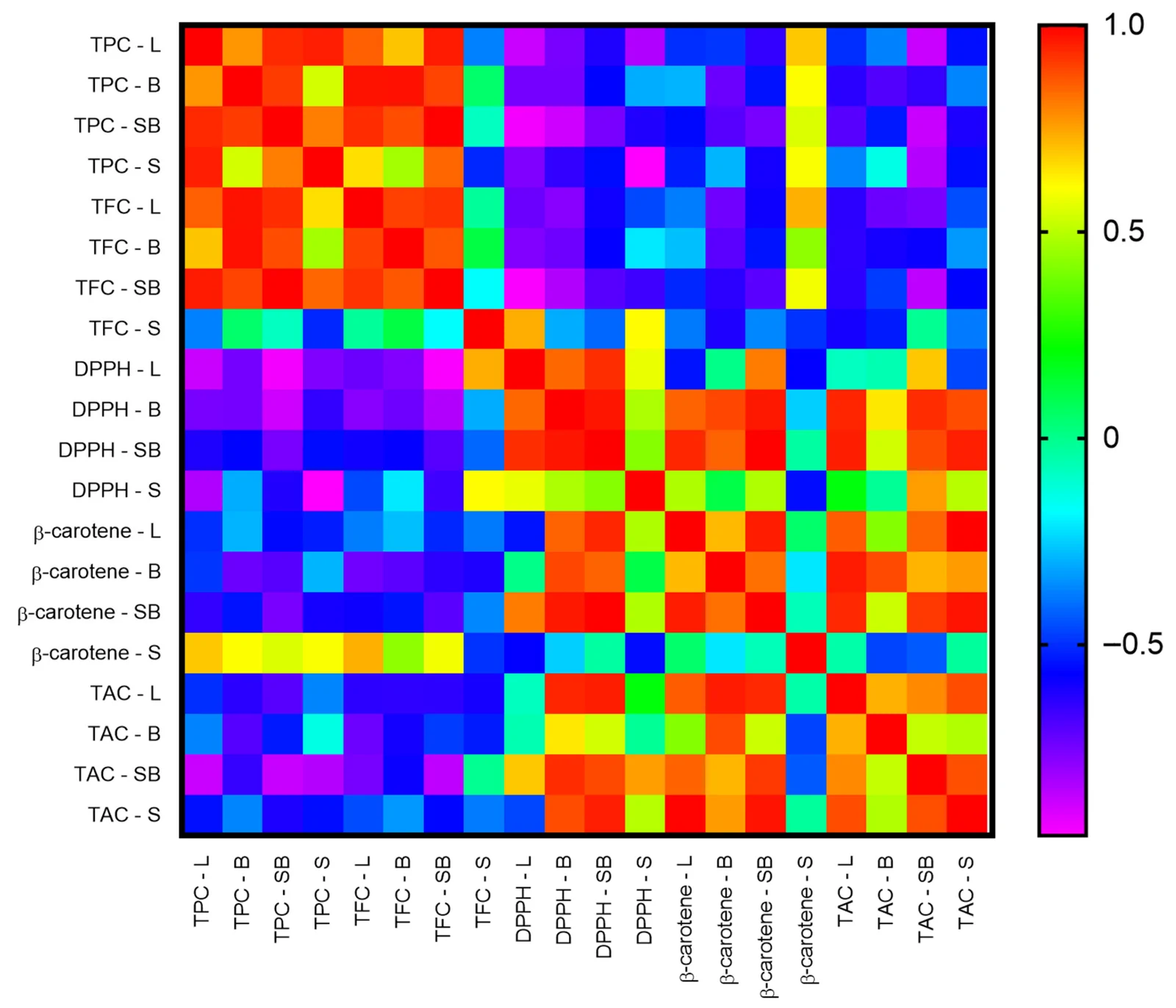
Heat map of correlation coefficients between TPC, TFC, and antioxidant assays in *E. nummularium*.

A correlation analysis was conducted to investigate the relationships between TPC, TFC, and antioxidant activity, as assessed by the DPPH, β-carotene bleaching, and TAC assays. Normality was evaluated using the Shapiro–Wilk test and visual inspection of Q–Q plots. Depending on the distribution of each variable, Pearson’s or Spearman’s correlation coefficients were applied. The results are visualized in a heat map (Figure 1). Where positive correlations are represented in red and negative correlations in blue. The intensity of each color reflects the strength of the correlation coefficient. To facilitate interpretation, correlation strength was classified according to the following criteria: ±0.01 ≤ r < ±0.10 = very weak; ±0.10 ≤ r < ±0.40 = weak; ±0.40 ≤ r < ±0.70 = moderate; ±0.70 ≤ r < ±0.90 = strong; ±0.90 ≤ r < ±1.00 = very strong, and r = ±1.00 = perfect [45,46].

Strong to very strong positive correlations were observed between TPC and TFC across most plant organs, except for the stem, where the correlation with TFC was notably weaker. The most significant positive correlations included: Stem bark TPC and stem bark TFC (r = 0.9957, *p* ≤ 0.0001); Branch TPC and branch TFC (r = 0.9739, *p* ≤ 0.05); Branch TPC and leaf TFC (r = 0.9726, *p* ≤ 0.05).

In the analysis of DPPH assay results versus TPC/TFC, moderate, strong, very strong, and negative correlations were observed, except for the stem TFC, which showed no significant association. The most notable correlations were: Stem DPPH and stem TPC (r = −0.963, *p* ≤ 0.05); Leaf DPPH and stem bark TFC (r = −0.9573, *p* ≤ 0.05); Leaf DPPH and stem bark TPC (r = −0.9453, *p* ≤ 0.05).

For the β-carotene bleaching assay, weak, moderate, and strong correlations were observed, predominantly negative. Exceptions were noted in the stem, where correlations were less pronounced. The most relevant associations included: Stem bark β-carotene and stem bark TPC (r = −0.7547); Branch β-carotene and leaf TFC (r = −0.7416), and branch β-carotene and branch TPC (r = −0.7370).

Regarding TAC, all correlations with TPC and TFC were negative, ranging from weak to strong. The most significant were: Stem bark TAC and leaf TPC (r = −0.8803); stem bark TAC and Stem bark TPC (r = −0.8778), and stem bark TAC and Stem bark TFC (r = −0.8622).

### 3.7. Gas Chromatography Coupled to Mass Spectrometry (GC-MS)

The GC–MS Post-Run Analysis Program was employed to process the raw spectral data. Compounds identified in the derivatized extracts are listed in the Supplementary Materials (Table S1). Identification was based on fragmentation patterns observed in the mass spectra, matched against an integrated spectral database with similarity indices exceeding 90%, and further corroborated by literature references. Quantification was performed by calculating the relative percentage of each peak area, with major compounds defined as those presenting the highest area percentages.

The analysis revealed the presence of palmitic acid, β-sitosterol, methyl linoleate, methyl hexadecanoate, linoleic acid, and lupeol acetate across multiple plant organs, including leaves, branches, stem bark, and stem.

In the DLEN fraction, the predominant compounds were glycerol (13.35%), palmitic acid (8.51%), and glyceryl palmitate (7.18%). The HLEN fraction was characterized by high levels of palmitic acid (23.67%) and methyl hexadecanoate (16.58%).

In the DBEN fraction, glycerol (5.31%), methyl 3,4-dihydroxybenzoate (2.75%), and malic acid (2.63%) were the most abundant. The HBEN fraction contained methyl hexadecanoate (16.59%), lupeol acetate (11.41%), and palmitic acid (9.99%) as major constituents.

For the DSBEN fraction, the predominant compounds were methyl octadecanoate (19.93%), methyl hexadecanoate (16.14%), and (9Z)-octadec-9-enoic acid (9.27%). As for the HSBEN fraction, the major compounds were methyl (E)-octadec-11-enoate (19.93%), methyl hexadecanoate (16.14%), and trans-9-Octadecenoic acid (9.27%).

In the DSEN fraction, the most abundant compounds were elaidic acid (15.93%), (11E)-octadec-11-methyl enoate (7.72%), and β-sitosterol (7.59%).

### 3.8. Antibacterial Activity

Antibacterial assays were conducted using various extracts and fractions of *E. nummularium* against *Streptococcus mutans*, *Streptococcus sobrinus*, and *Staphylococcus aureus*, including both ATCC reference strains and clinical isolates (Table 9 and Table 10). Minimum inhibitory concentrations (MICs) were determined to assess antibacterial efficacy.

For *S. mutans*, among the tested fractions, MSBEN, DSBEN, and ESBEN exhibited notable activity against *S. mutans*. MSBEN showed MIC values of 1000 µg/mL against both ATCC 700610 and ATCC 25175. DSBEN and ESBEN demonstrated stronger inhibition against ATCC 700610 (MIC = 500 µg/mL), while maintaining moderate activity against ATCC 25175 (MIC = 1000 µg/mL). No fraction exhibited significant inhibition against *S. sobrinus* at the tested concentrations.

The results for *S. aureus* were more promising, particularly against clinical isolates. The DLEN fraction displayed the highest efficacy, with an MIC of 62.5 µg/mL for isolates 29 and 92. Similarly, the ELEN and BLEN fractions showed comparable MIC values, reinforcing their potential. The DBEN fraction also demonstrated a MIC of 62.5 µg/mL, albeit against isolate 16A. MLEN and HLEN exhibited moderate activity, with MICs of 125 µg/mL (isolate 29) and 250 µg/mL (isolate 92). MBEN showed activity against isolate 16A (MIC = 500 µg/mL), while DSBEN inhibited ATCC 6538 and isolates 112 and 92 (MIC = 500 µg/mL). ESBEN and BSBEN also showed relevant activity against isolates 92 and 112, respectively (MIC = 500 µg/mL).

Overall, the crude methanolic extracts generally exhibited lower antibacterial activity compared to their respective fractions. In contrast, partitioned fractions (especially those obtained with dichloromethane and butanol) demonstrated enhanced efficacy, particularly against *S. aureus* clinical isolates.

### 3.9. Toxicity to Artemia salina Leach

The toxicity assay using A. salina allowed for the determination of the median lethal dose (LD_50_) of methanolic extracts and solvent-partitioned fractions obtained from different organs of *E. nummularium*. According to established criteria [47,48], LD_50_ values were classified as: highly active (<100 µg·mL^−1^), moderately active (100–500 µg·mL^−1^), weakly active (501–1000 µg·mL^−1^), and inactive (>1000 µg·mL^−1^) (Table 11).

The crude methanolic extract of the branches exhibited highly active toxicity (LD_50_ = 91.24 µg·mL^−1^). In contrast, extracts from the stem bark, stem, and leaves showed weak activity, with LD_50_ values of 227.3, 254.8, and 466.2 µg·mL^−1^, respectively.

Among the fractions, hexane extracts demonstrated the highest toxicity, being classified as highly active for branches (LD_50_ = 19.8 µg·mL^−1^) and stem bark (LD_50_ = 14.29 µg·mL^−1^), and moderately active for leaves (LD_50_ = 124.1 µg·mL^−1^).

Dichloromethane fractions also showed high activity for branches (LD_50_ = 94.77 µg·mL^−1^) and stem bark (LD_50_ = 81.31 µg·mL^−1^), and moderate activity for leaves (LD_50_ = 178.8 µg·mL^−1^) and stem (LD_50_ = 373.0 µg·mL^−1^).

Ethyl acetate fractions were inactive for leaves, but showed moderate toxicity for stem bark (LD_50_ = 211.5 µg·mL^−1^), branches (LD_50_ = 306.2 µg·mL^−1^), and stem (LD_50_ = 355.0 µg·mL^−1^).

Butanol fractions demonstrated moderate activity for stem bark (LD_50_ = 100.6 µg·mL^−1^), branches (LD_50_ = 300.7 µg·mL^−1^), and stem (LD_50_ = 479.3 µg·mL^−1^), while the leaf fraction was weakly active (LD_50_ = 832.4 µg·mL^−1^).

### 3.10. Effects of ELEN Extract on Acetic Acid-Induced Abdominal Writhing

**Figure 2:**
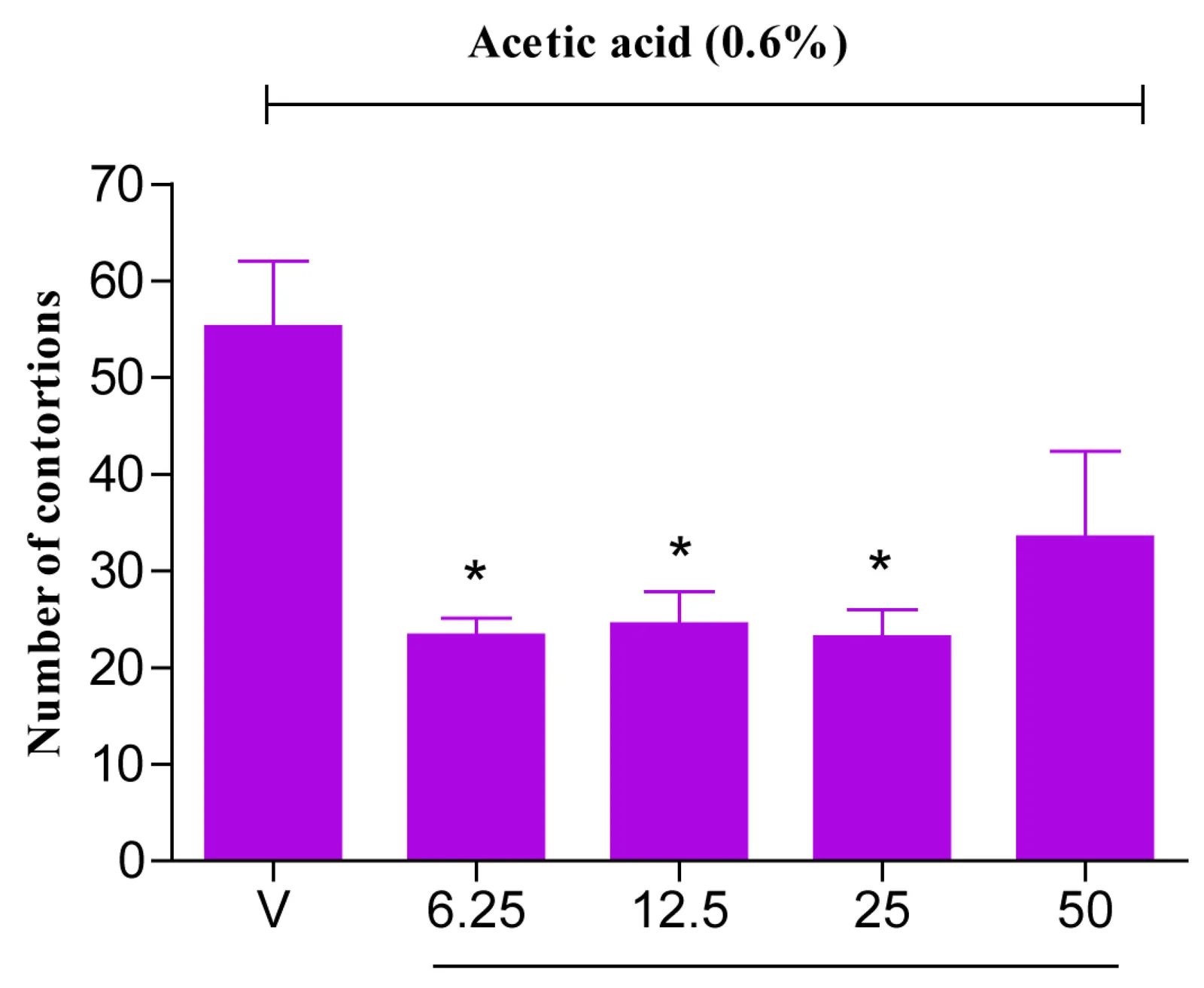
Antinociceptive activity of ELEN extract in the Acetic Acid-Induced Writhing Test. Animals were treated with doses of 6.25, 12.5, 25, and 50 mg/kg. V: Vehicle (control group). * Indicates a significant difference.

The antinociceptive potential of the ELEN fraction was evaluated using the acetic acid-induced abdominal writhing test in mice. The results are illustrated in Figure 2, which presents the mean number of writhes observed following administration of ELEN at doses of 6.25, 12.5, 25.0, and 50.0 µg·mL^−1^.

Significant reductions in the number of writhes were observed at doses of 6.25, 12.5, and 25.0 µg·mL^−1^ when compared to the control group (*p* < 0.05), indicating an apparent antinociceptive effect at these concentrations.

At the 50.0 µg·mL^−1^ dose, although a reduction in writhing was observed, the difference was not statistically significant relative to the control group. This may indicate a plateau or reversal in efficacy at higher concentrations.

These findings demonstrate that the ELEN fraction exhibits dose-sensitive antinociceptive activity, with greater effectiveness at lower doses, particularly between 6.25 and 25.0 µg/mL^−1^.

### 3.11. Acute Toxicity

The acute toxicity of the ELEN fraction was evaluated in mice following oral administration at a dose of 2000 mg·kg^−1^. Throughout the 15-day observation period, no mortality was recorded, and no clinical signs of toxicity, such as tremors, paralysis, piloerection, or cutaneous lesions, were observed in any of the treated animals.

At the end of the study, all animals were euthanized for hematological and macroscopic evaluation. Blood samples were collected for complete blood count (CBC) analysis, and the results are presented in Table S2, including mean values, standard deviations, and reference ranges.

While most hematological parameters remained within physiological limits, small deviations were observed in specific markers:

- Hemoglobin concentration (HGB) showed a reduction from 14.4 ± 7.8 g·dL^−1^ in the control group to 11.6 ± 3.96 g·dL^−1^ in the treated group, indicating a mild decrease in oxygen-carrying capacity.
- Red cell distribution width (RDW%) increased from 14.7 ± 1.1% (control) to 16.93 ± 0.25% (treated), suggesting greater anisocytosis and possible early erythropoietic stress.
- Mean platelet volume (MPV) rose from 6.2 ± 1.2 fL (control) to 7.23 ± 0.31 fL (treated), exceeding the reference range and potentially reflecting changes in platelet activation or turnover.

Other parameters, including white blood cell (WBC) count, red blood cell (RBC) count, and platelet count (PLT), remained within expected physiological ranges.

Macroscopic examination of vital organs (liver, kidneys, spleen, heart, lungs) revealed no visible pathological changes, reinforcing the absence of systemic toxicity at the tested dose.

Taken together, these findings suggest that the ELEN fraction is well tolerated at 2000 mg·kg^−1^, although subtle hematological changes, particularly in HGB, RDW%, and MPV, were observed.

## 4. Discussion

As shown in Table 1, the ethyl acetate and butanol fractions consistently yield the highest yields, particularly from the stem bark and leaves. This outcome aligns with the polarity profile of phenolics, flavonoids, glycosylated flavonoids, and tannins, which are preferentially extracted by intermediate to polar solvents due to their hydrophilic nature and solubility properties [49].

Qualitative phytochemical screening (Table 2) revealed a widespread occurrence of secondary metabolites across the fractions of *E. nummularium*. Phenolic compounds were detected in all samples, while flavonoids were absent only in the hexane fraction of the leaves and the butanol fraction of the stem. Saponins, steroids, and tannins were also frequently identified across various plant organs and solvent partitions, reflecting the genus’s well-documented chemical diversity. These findings are consistent with previous reports highlighting the presence of flavonoids, alkaloids, phenolic derivatives, and other bioactive constituents in *Erythroxylum* species [12,13] and reinforce the pharmacological and multidisciplinary potential of *E. nummularium* as a source of therapeutically relevant metabolites.

Quantitative analysis of total alkaloid content (TAT) further corroborated the findings from the preliminary phytochemical screening. As presented in Table 3, the highest concentrations of alkaloids were quantified in the hexane fractions of branches (HBEN) and stem bark (HSBEN), as well as in the dichloromethane fraction of leaves (DLEN), with values ranging from 22.67 to 24.03 mg EA g^−1^. These results suggest that a subset of alkaloids present in *E. nummularium* may possess moderate lipophilicity, thereby favoring extraction by solvents of lower polarity. The absence of detectable alkaloids in the ethyl acetate and butanol fractions is consistent with the negative outcomes of the qualitative assays. It reinforces the solvent-dependent selectivity of alkaloid extraction [50].

Following this preliminary phytochemical screening, the study proceeded to the quantification of total phenolics and flavonoids, as well as the evaluation of antioxidant activity. In this context, the antioxidant potential of *Erythroxylum nummularium* was assessed through multiple assays—DPPH, β-carotene/linoleic acid, and total antioxidant capacity (TAC) and correlated with total phenolic content (TPC) and total flavonoid content (TFC). The results revealed distinct antioxidant profiles across fractions and plant organs.

Among the organic phases, the hexane fraction consistently exhibited the highest EC_50_ values, indicating low antioxidant activity. This outcome aligns with the known polarity-dependent solubility of antioxidant compounds, as phenolics and flavonoids are typically polar and poorly extracted by nonpolar solvents, such as hexane [51,52]. Although antioxidant activity studies within the genus *Erythroxylum* remain limited [9,15,17,53,54,55,56,57], investigations involving *E. suberosum*, *E. cuneatum*, *E. coca*, and *E. macrocarpum* have reported findings consistent with our results. In these studies, polar fractions demonstrated superior antioxidant performance [9,15,17,54], reinforcing the relevance of solvent polarity in guiding the extraction of bioactive compounds from *Erythroxylum* species. In line with these results, the ethyl acetate and butanol fractions exhibited notably strong antioxidant activity, particularly in the leaves and stem bark, suggesting a higher concentration of bioactive polar compounds in these tissues. The dichloromethane fraction, although less polar, also showed promising results in leaves and branches, though its activity was less pronounced in the stem and bark. This result highlights that intermediate polarity solvents are also efficient in extracting some constituents with antioxidant potential [58]. This trend is consistent with phytochemical principles and reinforces the strategic use of solvent polarity to selectively isolate distinct classes of bioactive compounds [55] in *E. nummularium*.

These solvent-dependent extraction patterns become even more evident when the antioxidant activities of the fractions are compared to those of well-established reference compounds. In the DPPH and *β*-carotene/linoleic acid assays, ascorbic acid was employed as the standard antioxidant. In contrast, gallic acid and quercetin served as benchmarks in the total antioxidant capacity (TAC) assay. Remarkably, several fractions exhibited antioxidant activities that were statistically equivalent to these standards, underscoring the potent bioactivity of the phytochemicals present in *E. nummularium*. In the DPPH assay, the EBEN and MSBEN fractions showed no significant difference in activity compared to ascorbic acid. In the TAC assay, the ESBEN fraction demonstrated antioxidant capacity statistically indistinguishable from gallic acid, the most potent standard employed, while the EBEN and BSBEN fractions exhibited comparable activity to quercetin. Notably, in the β-carotene/linoleic acid assay, a broader range of fractions, including DLEN, ELEN, BLEN, MBEN, EBEN, ESBEN, and BSBEN, outperformed the standard, suggesting enhanced inhibition of lipid peroxidation. These findings are consistent with reports in the literature indicating that plant extracts rich in phenolic compounds, particularly those containing gallic acid and flavonoids, can exhibit antioxidant activities comparable to or even exceeding those of isolated standards [59,60]. Such results reinforce the concept that synergistic interactions among phytochemicals within complex plant matrices may contribute to elevated antioxidant efficacy, often surpassing the effects of individual compounds [61].

The heat map analysis (Figure 1) revealed that while strong and very strong positive correlations between TPC and TFC were consistently observed in most organs, particularly in stem bark and branches, the stem exhibited a notably weaker association. This divergence may reflect tissue-specific metabolic allocation or differential biosynthetic regulation of flavonoids in the stem, as previously reported in other species where lignified tissues prioritize structural phenolics over flavonoid accumulation [62].

The correlation patterns between antioxidant assays and TPC/TFC were equally insightful. In the DPPH assay, strong to very strong negative correlations were observed, most notably between stem DPPH and stem TPC (*r* = −0.963), leaf DPPH and stem bark TFC (*r* = −0.957), and leaf DPPH and stem bark TPC (*r* = −0.945). These associations indicate that phenolic-rich fractions contribute more decisively to radical scavenging capacity than those dominated by flavonoids. This observation aligns with the well-established antioxidant mechanisms of phenolic acids, which generally exhibit superior electron-donating ability and enhanced free radical stabilization compared to flavonoids under DPPH assay conditions [63].

In the *β*-carotene bleaching assay, weak to strong correlations were observed, predominantly negative. These negative correlations are consistent with the expected behavior of antioxidant systems, wherein lower EC_50_ values correspond to greater antioxidant efficacy. The most pronounced associations between stem bark *β*-carotene and stem bark TPC (*r* = −0.755), branch β-carotene and leaf TFC (*r* = −0.742), and branch β-carotene and branch TPC (*r* = −0.737), suggest that phenolic- and flavonoid-rich fractions contribute to lipid peroxidation inhibition [64,65]. Although the *β*-carotene assay is typically more sensitive to lipophilic antioxidants, our results indicate that polar compounds present in *E. nummularium* retain appreciable activity in emulsion-based systems, likely due to their structural capacity to neutralize peroxyl radicals [66]. An exception was noted in the stem, where positive correlations were observed, reflecting a distinct antioxidant profile with reduced contributions from hydrophilic constituents.

In the TAC assay, all correlations with TPC and TFC were negative, ranging from weak to strong. The most pronounced associations were observed between stem bark TAC and leaf TPC, stem bark TPC, and stem bark TFC (*r* = −0.880, −0.878, and −0.862, respectively), suggesting that elevated concentrations of phenolic and flavonoid compounds are associated with enhanced total antioxidant capacity. This pattern is consistent with the redox behavior of these compounds, which can act through multiple electron-transfer mechanisms beyond radical scavenging alone, including metal chelation and synergistic interactions within complex phytochemical matrices [67].

Taken together, these correlation profiles underscore the importance of employing complementary antioxidant assays to capture the multifaceted nature of bioactivity [68]. They also reinforce the concept that phenolic and flavonoid compounds contribute differentially to antioxidant mechanisms, depending on their chemical structures, polarities, and tissue-specific distributions within the plant. Thus, we can guide targeted extraction strategies that aim to maximize the antioxidant potential of *E. nummularium*.

Following a comprehensive assessment of antioxidant activity and its correlation with TPC and TFC, the investigation proceeded to evaluate the antibacterial potential of *E. nummularium* extracts and their solvent-partitioned fractions. This phase aimed to explore the species’ capacity to inhibit the growth of clinically relevant oral and systemic pathogens. Antibacterial assays were conducted against *S. mutans*, *S. sobrinus*, and *S. aureus*. The antimicrobial efficacy of each sample was determined through minimum inhibitory concentration (MIC) and minimum bactericidal concentration (MBC) analyses, as detailed in Table 9 and Table 10.

The results revealed a selective and strain-dependent antibacterial profile across the tested fractions. In the case of *S. mutans*, the BLEN, DSBEN, and ESBEN fractions exhibited the most pronounced inhibitory effects. Notably, MSBEN, DSBEN, and ESBEN demonstrated activity against both ATCC strains; however, DSBEN and ESBEN were particularly effective against *S. mutans* ATCC 700610, suggesting a degree of strain-specific sensitivity. In contrast, none of the evaluated fractions exhibited detectable activity against *S. sobrinus* at the tested concentrations, suggesting potential intrinsic resistance or markedly reduced susceptibility to the phytochemicals of *E. nummularium*.

The lack of antibacterial activity against *S. sobrinus* may be attributed to structural and physiological differences between the tested streptococcal strains, including variations in cell wall polysaccharide architecture, membrane permeability, and potentially distinct regulatory or efflux mechanisms [69]. These differences may influence susceptibility to phytochemicals. Conversely, the moderate yet consistent activity observed against *S. mutans* suggests that specific fractions, particularly those derived from stem bark, may hold promise as preventive agents in the management of dental caries. This interpretation aligns with previous findings in the genus *Erythroxylum*. For instance, Loyola et al. [70] reported that ethanolic extracts of *E. coca* leaves inhibited *S. mutans* ATCC 25175, a result corroborated by the present study. To our knowledge, this is the first report to evaluate the antibacterial activity of *Erythroxylum* species against *S. sobrinus*, thereby expanding the genus’s microbiological scope and highlighting the need for further investigation of its antimicrobial spectrum.

Extending the antimicrobial assessment to systemic pathogens, the fractions of *E. nummularium* demonstrated a pronounced inhibitory effect against *Staphylococcus aureus*, particularly among clinical isolates. The dichloromethane (DLEN), ethyl acetate (ELEN), and butanol (BLEN) leaf fractions exhibited the most potent activity, with MIC values of 62.5 µg/mL against isolates 29 and 92. Similarly, the stem bark-derived DBEN fraction showed equivalent potency against isolate 16A. These MIC values fall within pharmacologically relevant thresholds, suggesting the presence of structurally diverse bioactive constituents with strong antibacterial potential. Additional fractions, including DBEN, MLEN, HLEN, MBEN, DSBEN, ESBEN, and BSBEN, also exhibited moderate to significant activity, reinforcing the antimicrobial richness of both leaf and stem bark extracts.

These findings are consistent with previous studies involving other *Erythroxylum* species. For instance, polar fractions of *E. caatingae*, specifically AcOEt:MeOH (90:10) and AcOEt:MeOH (95:5), exhibited MIC values of 250 µg/mL against *S. aureus* [18]. Similarly, methanolic extracts from the leaves and twigs of *E. macrocarpum* demonstrated MIC values of 500 µg/mL against the same pathogen [20]. In South African species such as *Erythroxylum delagoense*, *E. emarginatum*, and *E. pictum*, acetone and methanol extracts from both leaves and bark have also demonstrated inhibitory activity against *S. aureus*. Specifically, the acetone leaf extract of *E. delagoense* exhibited MIC values of 500 µg/mL, while higher MIC values of 1000 µg/mL were observed for the methanolic leaf extract and aqueous bark extract of the same species. Comparable MIC values were reported for the acetone and methanolic bark extracts of *E. emarginatum*, as well as for the acetone extracts of both leaves and bark of *E. pictum*. Although these results confirm the antimicrobial potential of various *Erythroxylum* species, the fractions evaluated in the present study, particularly DLEN, ELEN, BLEN, and DBEN, demonstrated markedly superior efficacy, with MIC values four to sixteen times lower than those previously reported. These fractions exhibited minimum inhibitory concentrations as low as 62.5 µg/mL against clinical isolates of *S. aureus*, underscoring the high antibacterial potential of *E. nummularium*.

The enhanced activity of DLEN, ELEN, BLEN, and DBEN may be attributed to their phytochemical composition. These fractions are enriched in flavonoids, tannins, and phenolic compounds, classes of secondary metabolites widely recognized for their antimicrobial properties [20,71,72]. Notably, tannins have been shown to inhibit bacterial growth and biofilm formation, with evidence suggesting that their activity may involve interactions with bacterial membranes and surface structures [73]. The presence of these compounds in *E. nummularium* fractions aligns with the metabolomic profiles observed in *E. mexicanum*, where terpenoids, alkaloids, and phenolics predominated in the most active antibacterial fractions [74].

Interestingly, the HLEN extract also exhibited measurable antibacterial activity against several tested strains, despite its relatively low content of phenolic and flavonoid compounds. This observation suggests that nonpolar constituents, such as fatty acids, terpenoids, or other lipophilic metabolites, may play a significant role in the observed antimicrobial effects. Such a hypothesis is supported by previous findings in *E. mexicanum*, where hexane fractions rich in diterpenoids demonstrated potent antibacterial activity, with MIC values of 62.5 µg/mL reported against both *S. aureus* and *Streptococcus pyogenes* [74]. These results highlight the importance of nonpolar phytochemical classes in contributing to antibacterial efficacy and reinforce the understanding that bioactivity in *Erythroxylum* species is not solely dependent on polar constituents such as flavonoids and tannins.

Taken together, these findings support the hypothesis that *E. nummularium* harbors a structurally diverse array of pharmacologically active metabolites capable of inhibiting *S. aureus*, including multidrug-resistant clinical strains. The demonstrated efficacy across both polar and nonpolar fractions reinforces the therapeutic relevance of this species and underscores its potential as a source of novel antimicrobial agents. Further investigations aimed at isolating, structurally characterizing, and mechanistically evaluating its bioactive constituents are warranted and may contribute meaningfully to the development of plant-derived alternatives in antimicrobial therapy.

The cytotoxic potential of methanolic extracts from various organs of *E. nummularium*, along with their solvent-partitioned fractions, was assessed using the *Artemia salina* lethality bioassay, a widely recognized preliminary model for evaluating general toxicity and bioactivity. The *A. salina* lethality assay was used as a preliminary, broad-spectrum toxicity screen to prioritize fractions for further in vivo toxicity investigation. This assay provides a sensitive, cost-effective platform for detecting the toxic effects of natural products, particularly those with potential cytotoxic and antitumor properties [75].

In this study, hexane fractions exhibited the highest toxicity among all samples tested, with LD_50_ values ranging from 14.29 to 124.1 µg mL^−1^, indicating high or moderate toxicity according to established thresholds [47,48]. Low LD_50_ values are frequently indicative of compounds with cytotoxic potential, as there is a well-documented correlation between high toxicity and low LD_50_ values [76,77].

Rajabi et al. (2015) demonstrated the validity of the *A. salina* assay by comparing it with the MTT assay in multiple cell lines, reporting no statistically significant differences (*p* > 0.05) between the two methods [78]. This reinforces the utility of *A. salina* as a rapid, cost-effective alternative for preliminary cytotoxicity screening. In our study, all crude methanolic extracts and fractions of *E. nummularium* exhibited measurable toxicity, except for ELEN, which was classified as inactive. A previous investigation demonstrated that although the methanolic extract of *E. nummularium* leaves was inactive in the brine shrimp lethality test, the isolated diterpene 6α,11α-epoxy-14α-methoxy-ryanodane-1α,5β,7β,11β,13β-pentaol, commonly known as 14-*O*-methyl-ryanodanol, exhibited marked toxicity, with an LD_50_ value of 21.8 µg·mL^−1^. This compound was also identified in *E. passerinum*, where it demonstrated larvicidal activity against *Aedes aegypti* and significant toxicity in the *A. salina* assay [22]. These findings underscore the importance of bioguided phytochemical isolation and suggest that *E. nummularium* may represent a relevant source of structurally distinctive and biologically active metabolites, warranting further studies in more specific pharmacological models.

Species within the *Erythroxylum* genus are well recognized for their antiproliferative and pro-apoptotic properties, as demonstrated across distinct cellular models. For instance, the hexane extract of *E. daphnites* significantly inhibited the proliferation of oral squamous cell carcinoma (SCC-9) cells by inducing G1 cell cycle arrest, downregulating cyclins D and E, and promoting apoptosis via caspase-3 activation [79]. Similarly, the diterpene 14-*O*-methyl-ryanodanol, isolated from *E. passerinum*, exhibited dose-dependent cytotoxicity in astrocytic GL-15 cells, with nuclear condensation, DNA fragmentation, and apoptotic body formation [80]. In a related study, *E. macrocalyx* was shown to produce structurally diverse tropane alkaloids, among which compound 5 demonstrated potent antiproliferative activity against HepG2 hepatocellular carcinoma cells (IC_50_ = 3.66 µg·mL^−1^), while sparing non-neoplastic lymphoblasts [81]. These findings collectively highlight the chemical diversity of secondary metabolites from the genus *Erythroxylum* and their promising anticancer potential.

Additional evidence supports the cytotoxic potential of tropane alkaloids isolated from *Erythroxylum* species. Catuabin B, isolated from *E. caatingae*, exhibited cytotoxicity against K562 (chronic myeloid leukemia) cells and induced apoptosis [18]. Likewise, erythrobezerrine C from *E. bezerrae* showed moderate cytotoxicity against HCT-116 (colon adenocarcinoma) and NCI-H460 (lung cancer) cell lines [82].

Collectively, these findings suggest that *E. nummularium* is a promising candidate for further cytotoxic investigations, particularly given its capacity to biosynthesize alkaloids, flavonoids, and diterpenoids, classes of compounds with well-established anticancer properties [83,84,85]. Isolation and structural elucidation of these metabolites are essential for understanding the mechanisms underlying their toxicity. Moreover, the lack of toxicity observed for ELEN (LD_50_ > 1000 µg·mL^−1^) suggests its potential safety for in vivo biological assays.

Among the tested fractions, ELEN showed high total phenolic and flavonoid contents, relevant antioxidant activity, potent antibacterial activity against *S. aureus*, and low toxicity in the *Artemia salina* lethality assay. Thus, ELEN was selected because it uniquely combined high phenolic and flavonoid content, strong antioxidant activity, potent antibacterial activity against *S. aureus*, and low preliminary toxicity. This integrated profile made ELEN the most promising and ethically appropriate candidate for the in vivo antinociceptive and acute oral toxicity studies. In this context, the present study also provides the first in vivo evidence of the antinociceptive activity of an *E. nummularium* fraction. The analgesic effect of ELEN was evaluated using the acetic acid-induced abdominal writhing test in mice, a widely accepted model for assessing peripheral nociception and the efficacy of analgesic compounds. The mechanism of action involves the activation of nociceptors by acetic acid, leading to the release of pro-inflammatory mediators, such as prostaglandins and nitric oxide, which, in turn, stimulate pain perception and elicit protective behavioral responses.

The ELEN fraction exhibited significant antinociceptive activity, as evidenced by a marked reduction in abdominal writhing in the acetic acid-induced nociception model. Notably, the lowest dose tested (6.25 mg·kg^−1^) produced the most pronounced effect, reducing the number of writhes by 63%, compared to reductions of 56% and 57% observed at intermediate doses of 12.5 and 25 mg·kg^−1^, respectively. In contrast, the highest dose (50 mg·kg^−1^) did not yield a statistically significant difference relative to the vehicle control. This inverse dose–response relationship may reflect receptor saturation, reduced bioavailability, or the activation of compensatory signaling pathways, such as receptor desensitization and mitogen-activated protein kinase (MAPK) cascade engagement, at higher concentrations, warranting further pharmacokinetic and mechanistic investigation [86]. Furthermore, this inverse dose–effect relationship may occur due to the complex chemical composition of ELEN, suggesting that pro-nociceptive or counter-regulatory constituents of ELEN may become functionally relevant at higher doses, masking or reducing the antinociceptive effects observed at lower doses. Thus, the inverse dose–response pattern does not invalidate the significant antinociceptive activity observed at 6.25–25 mg/kg, but limits extrapolation to higher doses and highlights the need for more refined pharmacodynamic studies.

Previous phytochemical investigations of *E. nummularium* leaves have identified flavonoids, including epicatechin, quercetin, and their structural derivatives [21,23]. Among these, epicatechin and quercetin are particularly noteworthy due to their well-established antinociceptive and anti-inflammatory properties [87,88]. Epicatechin has been shown to exert antinociceptive effects through multiple mechanisms, including activation of the nitric oxide–cyclic GMP–ATP-sensitive K^+^ channel pathway and modulation of serotonergic (5-HT_1_A, 5-HT_1_B, 5-HT_1_D, and 5-HT_5_A) and opioid (μ, κ, δ) receptors, as demonstrated in inflammatory and neuropathic pain models [87]. Quercetin, in turn, displays multimodal analgesic activity by modulating neuronal excitability through inhibition of mechanosensitive ion channels (ASIC and TRPA1), voltage-gated sodium (Nav) and calcium (Cav) channels, and by facilitating potassium (Kv) channel activity. Additionally, it suppresses cyclooxygenase-2 (COX-2) expression and prostaglandin E_2_ synthesis, contributing to both local anesthetic and anti-inflammatory effects [88]. These mechanisms are consistent with the pharmacological profile of the ELEN fraction and suggest that its antinociceptive activity may, at least in part, be attributed to the presence of these bioactive flavonoids. This interpretation is further supported by recent studies demonstrating that polyphenolic compounds can attenuate nociceptive responses by suppressing pro-inflammatory mediators, including COX-2 and PGE_2_, and by modulating neuronal excitability through inhibition of peripheral sensitization mechanisms and reversal of inflammation-induced hyperexcitability in primary afferent and trigeminal wide-dynamic range neurons [89,90].

Furthermore, phenolic compounds commonly found in the *Erythroxylum* genus have been implicated in modulating inflammatory responses [11,12]. The antinociceptive properties demonstrated in this study are consistent with these findings and reinforce the therapeutic potential of ELEN as a source of analgesic agents.

In summary, the ELEN fraction demonstrates compelling antinociceptive potential, likely mediated by flavonoids such as epicatechin and quercetin. These findings support further pharmacological exploration and highlight *E. nummularium* as a promising candidate for the development of novel analgesic therapies.

The acute toxicity assessment of the ELEN fraction at a dose of 2000 mg·kg^−1^ revealed no mortality or overt clinical signs of toxicity throughout the 15-day observation period. Treated animals maintained normal physical and behavioral parameters, with no evidence of tremors, paralysis, aggression, or cutaneous lesions, suggesting a favorable safety profile at the tested dose. Macroscopic examination of vital organs (liver, kidneys, spleen, heart, lungs) revealed no visible pathological alterations, further supporting the absence of systemic toxicity.

Hematological analyses further supported these findings, with most parameters, including WBC, RBC, and PLT, remaining within established reference ranges. Nevertheless, minor deviations were observed in select indices, including a slight reduction in hemoglobin (HGB) concentration, a modest increase in red cell distribution width (RDW%), and a mild elevation in mean platelet volume (MPV). Although these changes did not reach statistical significance, they highlight the importance of more comprehensive toxicological assessments to evaluate potential cumulative or subclinical effects [91]. Long-term studies are therefore warranted to confirm the safety profile of the ELEN fraction and to ensure its suitability for therapeutic development.

The absence of macroscopic alterations in vital organs reinforces the low systemic toxicity of the ELEN fraction. These results are in line with previous reports demonstrating that plant-derived bioactive fractions typically exhibit low toxicity, a property attributed to their selective pharmacological activity and intrinsic biocompatibility, both of which result from their natural origin [92]. In this context, the observed reduction in HGB and the increases in RDW% and MPV in the treated animals may reflect transient physiological adaptations, rather than direct toxicological effects [93].

Taken together, these findings suggest that the ELEN fraction is well tolerated at 2000 mg·kg^−1^, with no evidence of acute systemic toxicity. Nonetheless, subtle hematological changes, particularly in HGB, RDW%, and MPV, warrant further investigation to ensure safety in long-term therapeutic applications. These data support the continued pharmacological development of ELEN as a promising bioactive extract with analgesic potential.

GC–MS was employed to characterize the chemical composition of dichloromethane and hexane fractions derived from various organs of *E. nummularium*. In contrast, more polar extracts, such as those obtained with ethyl acetate and butanol, were not subjected to GC–MS analysis due to their high polarity and low volatility, which limit direct detection by this technique. Therefore, a more comprehensive characterization of these fractions will require complementary chromatographic and spectrometric approaches, such as HPLC and LC–MS, which are better suited for analyzing the chemical profiles of the more polar constituents. The analysis revealed a chemically diverse profile comprising fatty acids, esters, phytosterols, and phenolic acids, many of which are recognized for their pharmacological relevance and favorable toxicological profiles. Among the most frequently identified compounds were palmitic acid, β-sitosterol, and lupeol acetate, which were consistently detected in both the dichloromethane and hexane fractions. In addition to these lipophilic constituents, several phenolic acid derivatives were identified in the dichloromethane fractions, notably 3,4-dihydroxybenzoic acid (protocatechuic acid), methyl 3,4-dihydroxybenzoate, and gallic acid. These compounds have been extensively documented for their anticancer, antinociceptive, anti-inflammatory, and antioxidant properties [94,95,96]. Their presence may contribute to the biological activities observed in vitro, particularly in assays evaluating antioxidant capacity.

Among all hexane and dichloromethane fractions obtained from the various organs of the studied plant species, the DSBEN fraction exhibited the highest total phenolic content and the most pronounced antioxidant activity in the DPPH assay. These results are consistent with its elevated concentrations of methyl 3,4-dihydroxybenzoate (7.49%) and gallic acid (6.62%), both of which are well-established phenolic antioxidants. The abundance of these compounds likely contributed to the enhanced radical-scavenging capacity observed in this fraction, as polyphenolic compounds are known to donate hydrogen atoms and stabilize free radicals through resonance delocalization [97].

The DLEN extract showed the highest antioxidant activity in the β-carotene bleaching assay among all fractions analyzed by GC-MS. This result can be directly correlated with its chemical composition, which includes a combination of phenolic acids, lipid derivatives, and oxygenated compounds known to contribute to radical-scavenging mechanisms. Notably, the presence of 3,4-dihydroxybenzoic acid (1.25%) and methyl 3,4-dihydroxybenzoate (1.98%) is of particular relevance. These phenolic acid derivatives are recognized for their ability to donate hydrogen atoms and stabilize free radicals through resonance delocalization, thereby interrupting lipid peroxidation chains in emulsified systems such as the β-carotene/linoleic acid model [98]. Furthermore, the methoxylation of the carboxylic group, as observed in methyl 3,4-dihydroxybenzoate, can increase lipophilicity and membrane permeability, improving interaction with lipid radicals and amplifying antioxidant efficacy. Moreover, the DLEN fraction contains linalool oxide (6.35%), a monoterpenoid with known antioxidant and anti-inflammatory properties that may act synergistically with phenolic acids to enhance radical-scavenging activity [99]. Glycerol (13.35%) may contribute indirectly by stabilizing the emulsion system and improving the solubilization of the active compounds. Although fatty acids and their derivatives, such as palmitic acid (8.51%) and 2,3-dihydroxypropyl hexadecanoate (7.18%), are not potent antioxidants, they can influence the polarity of the extract, facilitating the dispersion of more potent antioxidant compounds within the assay matrix.

In the total antioxidant capacity (TAC) assay, the ethyl acetate fraction from the stem bark (ESBEN) exhibited the highest overall activity. This fraction, however, was not subjected to GC–MS analysis due to its high polarity and low volatility, which limit direct detection by this technique. Its superior performance likely reflects enrichment in highly polar phenolics and flavonoids, compounds well known for their strong antioxidant potential. Among the fractions characterized by GC-MS, DBEN was the most active, with its chemical composition accounting for the antioxidant activity observed in this assay. Phenolic acids such as 4-hydroxy-3-methoxybenzoic acid and 3,4-dihydroxybenzoic acid, together with their derivative methyl 3,4-dihydroxybenzoate, are particularly relevant. These compounds are recognized for their antioxidant capacity. In addition to these phenolic constituents, DBEN contained several low-molecular-weight organic acids, including malic, succinic, oxalic, and glyceric acids, which may indirectly enhance antioxidant performance by modulating redox balance, chelating transition metals, and stabilizing reactive intermediates [100]. Our results reinforce the multifactorial nature of the antioxidant response, underscoring the synergistic interplay between phenolic acids and organic acids in shaping the overall antioxidant performance of *E. nummularium* extracts.

## 5. Conclusions

This study provides the first integrated assessment of the chemical composition and biological properties of methanolic extracts and fractions obtained from different organs of *Erythroxylum nummularium*. The extracts exhibited marked variation in total phenolic and flavonoid contents, as well as in metabolite profiles, which strongly influenced their antioxidant, antimicrobial, toxicological, and antinociceptive responses. Fractions enriched in polar phenolics, particularly ELEN and ESBEN, demonstrated superior antioxidant performance, while DSBEN, DLEN, and DBEN showed relevant activity among the fractions characterized by GC–MS. These findings reinforce the contribution of phenolic acids, flavonoids, and low-molecular-weight organic acids to the multifactorial mechanisms underlying the observed bioactivities.

Biologically, ELEN displayed notable antimicrobial activity against *Staphylococcus aureus*, low toxicity in *Artemia salina*, and significant antinociceptive effects in mice, supporting its relevance as a source of bioactive constituents. Although promising, these results represent preliminary evidence and should be interpreted within the limitations of the study, including the absence of chromatographic characterization of highly polar fractions and the exploratory nature of the in vivo assays.

Overall, the data highlight *E. nummularium* as a potential reservoir of structurally diverse and biologically active metabolites with antioxidant, antimicrobial, and antinociceptive properties. Future research should focus on chromatographic fractionation and isolation of individual compounds, structural elucidation of active constituents, mechanistic studies, and expanded in vivo pharmacological models to better define the therapeutic relevance of this species and support its potential application in natural product-based drug discovery.

## Figures and Tables

**Table 1:** Masses and codes of organic phases obtained by partitioning the crude MeOH extract of leaves, branches, stem bark, and stem of *E. nummularium*.

Plant Organs	Hexane (g)	Dichloromethane (g)	Ethyl Acetate (g)	Butanol (g)
Leaves	HLEN 0.893 g	DLEN 1.453 g	ELEN 15.241 g	BLEN 17.494 g
Branches	HBEN 2.783 g	DBEN 2.556 g	EBEN 27.968 g	BBEN 2.316 g
Stem bark	HSBEN 2.013 g	DSBEN 0.968 g	ESBEN 51.205 g	BSBEN 26.942 g
Stem	*	DSEN 1.102 g	ESEN 9.543 g	BSEN 52.000 g

* Fraction not obtained.

**Table 2:** Qualitative phytochemical analysis of *E. nummularium* fractions.

Fractions	Alkaloids	Flavonoids	Phenolics	Saponins	Steroids	Tannins
HLEN	+	−	+	+	+	+
DLEN	+	+	+	−	−	+
ELEN	−	+	+	−	+	+
BLEN	−	+	+	−	+	−
HBEN	+	+	+	+	+	+
DBEN	−	+	+	−	−	+
EBEN	−	+	+	−	+	+
BBEN	−	+	+	+	+	−
HSBEN	+	+	+	+	+	+
DSBEN	+	+	+	−	−	+
ESBEN	−	+	+	−	−	+
BSBEN	−	+	+	+	+	−
DSEN	+	+	+	+	+	−
ESEN	−	+	+	−	−	+
BSEN	−	−	+	−	+	−

**Table 3:** Total alkaloid content (TAT) expressed in mg EA g^−1^ of the various organs of *E. nummularium*.

Extract, Phases, and Pattern/Plant Organ	Leaves mg EA g^−1^ ± DP *	Branches mg EA g^−1^ ± DP *	Stem Bark mg EA g^−1^ ± DP *	Stem mg EA g^−1^ ± DP *
Hexane	18.78 ± 0.42 ^c^	24.03 ± 0.80 ^b^	23.12 ± 0.90 ^a,b^	#
Dichloromethane	22.67 ± 0.88 ^a,b^	#	21.24 ± 0.63 ^a,d^	20.03 ± 0.22 ^c,d^
Ethyl acetate	#	#	#	#
Butanol	#	#	#	#

* Results are expressed as mean ± Standard Deviation (*n* = 3). Values followed by the same letter showed no significant difference when comparing the values of the phases, *p* < 0.05 (One-way analysis of variance, followed by Tukey’s post hoc test). # Analysis was not performed for this sample due to a negative result in the preliminary qualitative assay. mg EA g^−1^ = milligrams of atropine equivalent per gram of extract.

**Table 4:** Total phenolic content (TPC) of *E. nummularium* organs, expressed as mg GAE·g^−1^.

Extract, Phases, and Pattern/Plant Organ	Leaves mg EAG g^−1^ ± DP *	Branches mg EAG g^−1^ ± DP *	Stem Bark mg EAG g^−1^ ± DP *	Stem mg EAG g^−1^ ± DP *
Crude	449.1 ± 13.7 ^f^	796.9 ± 26.2 ^i,j^	831.0 ± 26.2 ^j,l^	87.1 ± 3.2 ^b^
Hexane	13.3 ± 0.07 ^a^	0.0	0.0	#
Dichloromethane	35.2 ± 1.5 ^a^	22,6 ± 0,8 ^a^	183.9 ± 6.3 ^c^	6.8 ± 0.3 ^a^
Ethyl acetate	854.3 ± 29.9 ^l^	758.9 ± 24.1 ^i^	958.9 ± 23.9 ^m^	273.8 ± 11.2 ^e^
Butanol	591.0 ± 18.2 ^g^	217.3 ± 7.2 ^c,d^	658.9 ± 21.4 ^h^	247.6 ± 8.7 ^d,e^

* Values are expressed as mean ± standard deviation (*n* = 3). Means followed by the same letter do not differ significantly (*p* < 0.05), according to one-way ANOVA followed by Tukey’s post hoc test. Fractions with undetectable phenolic content were excluded from statistical analysis. # Not performed due to negative results in the qualitative assay. EAG g^−1^ = mg gallic acid equivalent per gram of extract.

**Table 5:** Total flavonoid content (TFC) of *E. nummularium* organs, expressed as mg QE·g^−1^.

Extract, Phases, and Pattern/Plant Organ	Leaves mg EQ g^−1^ ± DP *	Branches mg EQ g^−1^ ± DP *	Stem Bark mg EQ g^−1^ ± DP *	Stem mg EQ g^−1^ ± DP *
Crude	402.1± 5.6 ^g^	557.1 ± 13.4 ^i^	632.9 ± 13.9 ^j^	55.4 ± 1.7 ^b,c^
Hexane	43.1 ± 1.6 ^b^	34.8 ± 1.0 ^a,b^	37.9 ± 1.8 ^b^	#
Dichloromethane	85.8 ± 3.1 ^d,e^	43.8 ± 1.4 ^b^	110.7 ± 4.3 ^e^	74.5 ± 1.8 ^c,d^
Ethyl acetate	512.9 ± 18.0 ^h^	398.4 ± 4.4 ^g^	764.4 ± 18.6 ^l^	8.9 ± 0.5 ^a^
Butanol	179.3 ± 4.4 ^f^	197.7 ± 0.7 ^f^	542.3 ± 15.8 ^i^	0.0

* Values are expressed as mean ± standard deviation (*n* = 3). Means followed by the same letter do not differ significantly (*p* < 0.05), according to one-way ANOVA followed by Tukey’s post hoc test. Fractions with undetectable flavonoid content were excluded from statistical analysis. # Not performed due to negative results in the qualitative assay. mg QE·g^−1^ = milligrams of quercetin equivalent per gram of extract.

**Table 6:** Antioxidant activity of *E. nummularium* organs determined by DPPH assay.

Extract, Phases, and Pattern/Plant Organ	Leaves EC_50_ µg/mL ± DP *	Branches EC_50_ µg/mL ± DP *	Stem Bark EC_50_ µg/mL ± DP *	Stem EC_50_ µg/mL ± DP *
Crude	82.5 ± 3.5 ^c^	57.9 ± 2.4 ^b^	38.6 ± 0.9 ^a^	>1000
Hexane	>1000	>1000	>1000	#
Dichloromethane	867.3 ± 16.1 ^i^	335.3 ± 7.8 ^h^	128.4 ± 2.5 ^e^	>1000
Ethyl acetate	64.4 ± 2.5 ^b^	23.0 ± 0.9 ^a^	69.4 ± 1.8 ^b,c^	170.3 ± 6.3 ^f^
Butanol	101.6 ± 2.6 ^d^	213.5 ± 5.2 ^g^	66.3 ± 1.2 ^b,c^	185.4 ± 7.3 ^f^
Ascorbic acid	33.2 ± 0.2 ^a^			

* Values are expressed as mean ± standard deviation (*n* = 3). Means followed by the same letter do not differ significantly (*p* < 0.05), according to one-way ANOVA followed by Tukey’s post hoc test. Inactive extracts were excluded from statistical analysis. # Not carried out.

**Table 7:** Antioxidant activity of *E. nummularium* organs determined by β-carotene assay.

Extract, Phases, and Pattern/Plant Organ	Leaves EC_50_ µg/mL ± DP *	Branches EC_50_ µg/mL ± DP *	Stem Bark EC_50_ µg/mL ± DP *	Stem EC_50_ µg/mL ± DP *
Crude	108.8 ± 6.3 ^b,c,d,e^	68.3 ± 4.0 ^a,b,c,d^	113.7 ± 6.0 ^c,d,e^	223.3 ± 12.8 ^g,h^
Hexane	266.6 ± 10.2 ^h^	370.5 ± 18.5 ^i^	961.1 ± 63.3 ^l^	#
Dichloromethane	42.2 ± 2.3 ^a^	129.9 ± 1.3 ^d,e^	145.9 ± 2.3 ^e,f^	143.4 ± 2.6 ^e,f^
Ethyl acetate	65.4 ± 4.5 ^a,b,c^	90.2 ± 6.4 ^a,b,c,d,e^	57.4 ± 3.1 ^a,b,c^	442.4 ± 21.9 ^j^
Butanol	48.6 ± 2.4 ^a,b^	243.4 ± 6.1 ^g,h^	68.8 ± 3.9 ^a,b,c,d^	195.9 ± 2.3 ^f,g^
Ascorbic acid	201.5 ± 11.7 ^f,g^			

* Values are expressed as mean ± standard deviation (*n* = 3). Means followed by the same letter do not differ significantly (*p* < 0.05), according to one-way ANOVA followed by Tukey’s post hoc test. # Not carried out.

**Table 8:** Total Antioxidant Capacity (TAC) of *E. nummularium* organs assessed by phosphomolybdenum complexation.

Extract, Phases, and Pattern/Plant Organ	Leaves EC_50_ µg/mL ± DP *	Branches EC_50_ µg/mL ± DP *	Stem Bark EC_50_ µg/mL ± DP *	Stem EC_50_ µg/mL ± DP *
Crude	159.8 ± 4.3 ^c,d^	154.8 ± 7.0 ^c,d^	181.9 ± 0.7 ^d,e,f^	462.8 ± 14.7 ^j^
Hexane	469.9 ± 24.5 ^j^	250.7 ± 6.8 ^g,h^	379.5 ± 2.7 ^i^	#
Dichloromethane	198.5 ± 1.9^,e,f^	170.3 ± 8.0 ^d,e^	213.8 ± 3.0 ^f,g^	385.2 ± 18.3 ^i^
Ethyl acetate	199.6 ± 7.7 ^e,f^	128.3 ± 1.2 ^b,c^	61.8 ± 2.7 ^a^	393.6 ± 17.7 ^i^
Butanol	263.4 ± 8.3 ^h^	261.7 ± 5.4 ^h^	126.9 ± 3.0 ^b,c^	398.8 ± 19.9 ^i^
Gallic acid	37.8 ± 0.2 ^a^			
Quercetin	110.3 ± 3.4 ^b^			

* Values are expressed as mean ± standard deviation (*n* = 3). Means followed by the same letter do not differ significantly (*p* < 0.05), according to one-way ANOVA followed by Tukey’s post hoc test. # Not carried out.

**Table 9:** Antimicrobial activity of *E. nummularium* extracts and fractions against *S. mutans* and *S. sobrinus*.

	*S mutans* ATCC 700610		*S. mutans* ATCC 25175		*S. sobrinus* 6715	
	MIC (µg/mL)	MBC (µg/mL)	MIC (µg/mL)	MBC (µg/mL)	MIC (µg/mL)	MBC (µg/mL)
MLEN	**#**	**#**	1000	**#**	**#**	**#**
DLEN	#	#	#	#	#	#
HLEN	#	#	1000	#	#	#
ELEN	#	#	#	#	#	#
BLEN	500	#	#	#	#	#
MBEN	#	#	#	#	#	#
DBEN	#	#	#	#	#	#
HBEN	#	#	#	#	#	#
EBEN	#	#	#	#	#	#
BBEN	#	#	#	#	#	#
MSBEN	1000	#	1000	#	#	#
DSBEN	500	#	1000	#	#	#
HSBEN	#	#	#	#	#	#
ESBEN	500	#	1000	#	#	#
BSBEN	#	#	#	#	#	#
MSEN	#	#	#	#	#	#
DSEN	#	#	#	#	#	#
ESEN	#	#	#	#	#	#
BSEN	#	#	#	#	#	#

# There was no antimicrobial activity at the concentrations tested (31.25 to 1000 µg/mL).

**Table 10:** Antimicrobial activity of *E. nummularium* extracts and fractions against *S. aureus* (ATCC and Clinical Isolates).

	*S. aureus* ATCC		Isolated 16A		Isolated 112		Isolated 29		Isolated 92	
	MIC (µg/mL)	MBC (µg/mL)	MIC (µg/mL)	MBC (µg/mL)	MIC (µg/mL)	MBC (µg/mL)	MIC (µg/mL)	MBC (µg/mL)	MIC (µg/mL)	MBC (µg/mL)
MLEN	**#**	**#**	**#**	**#**	**#**	**#**	125	1000	125	1000
DLEN	#	#	#	#	#	#	62.5	1000	62.5	500
HLEN	#	#	1000	#	#	#	125	#	250	1000
ELEN	#	#	#	#	#	#	62.5	1000	62.5	500
BLEN	#	#	#	#	#	#	62.5	#	62.5	#
MBEN	#	#	500	#	#	#	#	#	#	#
DBEN	#	#	62.5	1000	#	#	#	#	#	#
HBEN	#	#	#	#	#	#	#	#	#	#
EBEN	#	#	#	#	#	#	#	#	#	#
BBEN	#	#	#	#	#	#	#	#	#	#
MSBEN	1000	#	#	#	1000	#	#	#	1000	#
DSBEN	500	#	#	#	500	#	#	#	500	#
HSBEN	#	#	#	#	#	#	#	#	#	#
ESBEN	1000	#	#	#	1000	#	#	#	500	#
BSBEN	#	#	#	#	500	#	#	#	#	#
MSEN	#	#	#	#	#	#	#	#	#	#
DSEN	#	#	#	#	#	#	#	#	#	#
ESEN	#	#	#	#	#	#	#	#	#	#
BSEN	#	#	#	#	#	#	#	#	#	#

# No antimicrobial activity was observed at the tested concentrations (31.25 to 1000 µg/mL).

**Table 11:** Median Lethal Dose (LD_50_) of *E. nummularium* extracts and fractions in *A. salina* toxicity assay.

Extract and Phases/Plant Organ	Leaves LD_50_ µg/mL	Branches LD_50_ µg/mL	Stem Bark LD_50_ µg/mL	Stem LD_50_ µg/mL
Methanolic extracts	466.2	91.24	227.3	254.8
Hexane	124.1	19.08	14.29	#
Dichloromethane	178.8	94.77	81.31	373.0
Ethyl acetate	Inactive	306.2	211.5	355.0
Butanol	832.4	300.7	100.6	479.3

# Toxicity assay not performed for this sample.

## Data Availability

Data are contained within the article.

## Supplementary Materials

The following supporting information can be downloaded at: https://www.mdpi.com/article/10.3390/antiox15081027/s1, Table S1. Chemical constituents identified by GC–MS in derivatized extracts of *E. nummularium*. Table S2. Hematological parameters after 15 days of treatment with the ELEN fraction (2000 mg·kg^−1^). Values are expressed as mean ± standard deviation (*n* = 6). Reference ranges are provided for comparison. Data were obtained using an automated hematological analyzer.

## Abbreviations

The following abbreviations are used in this manuscript:

ANOVA	Analysis of variance
ATP	Adenosine triphosphate
BBEN	Butanolic fraction of *E. nummularium* branches
β-carotene-L	β-carotene from leaves
β-carotene-B	β-carotene from branches
β-carotene-SB	β-carotene from stem bark
β-carotene-S	β-carotene from the stem
BHI	Brain Heart Infusion Agar
BLEN	Butanolic fraction of *E. nummularium* leaf
BSBEN	Butanolic fraction of the stem bark of *E. nummularium*
BSEN	Butanolic fraction of the stem of *E. nummularium*
DBEN	Dichloromethane fraction of *E. nummularium* branches
DLEN	Dichloromethane fraction of *E. nummularium* leaf
DMSO	Dimethyl sulfoxide
DP	Standard deviation
DPPH	2,2-diphenyl-1-picrylhydrazyl
DPPH-L	2,2-diphenyl-1-picrylhydrazyl from the leaves
DPPH-B	2,2-diphenyl-1-picrylhydrazyl from the branches
DPPH-SB	2,2-diphenyl-1-picrylhydrazyl from the stem bark
DPPH-S	2,2-diphenyl-1-picrylhydrazyl from the stem
DSBEN	Dichloromethane fraction of *E. nummularium* stem bark
DSEN	Dichloromethane fraction of the stem of *E. nummularium*
EBEN	Ethyl acetate fraction of *E. nummularium* branches
EC_50_	Efficient concentration at 50%
ELEN	Ethyl acetate fraction of *E. nummularium* leaf
ESBEN	Ethyl acetate fraction of *E. nummularium* stem bark
ESEN	Ethyl acetate fraction of *E. nummularium* stem
GC-MS	Gas chromatography coupled with mass spectrometry
HBEN	Hexane fraction of *E. nummularium* branches
HC	Hemoglobin concentration
HLEN	Hexane fraction of *E. nummularium* leaf
HSBEN	Hexane fraction of the stem bark of *E. nummularium*
LD_50_	Lethal dose
MBEN	Methanolic extract from the branches
MBC	Minimum bactericidal concentration
MEOH	Methanol
MIC	Minimum inhibitory concentration
µg	Micrograma
μL	Microlitro
MLEN	Methanolic extract of the leaf
μm	Micrometer
MPV	Mean platelet volume
MSBEN	Methanolic extract of stem bark
MSEN	Methanolic extract of the stem
M/Z	Mass charge
pH	Hydrogen ion potential
PLT	Platelet count
RBC	Red blood cell
RDW%	Red cell distribution width
TAC/CAT	Total alkaloid content/total antioxidant capacity
TAC-L	Total alkaloid content of the leaves
TAC-B	Total alkaloid content of the branches
TAC-SB	Total alkaloid content of the stem bark
TAC-S	Total alkaloid content of the stem
TFC	Total flavonoid content
TFC-L	Total flavonoid content of leaf
TFC-B	Total flavonoid content of branches
TFC-SB	Total flavonoid content of stem bark
TFC-S	Total flavonoid content of stem
TLC	Thin-layer chromatography analysis
TMCS	Trimethylchlorosilane
TPC	Total phenolic content
TPC-L	Total phenolic content of leaf
TPC-B	Total phenolic content of branches
TPC-SB	Total phenolic content of stem bark
TPC-S	Total phenolic content of stem
WBC	White blood cell

## References

[1] Bhatti J.S., Bhatti G.K., Reddy P.H. (2017). Mitochondrial Dysfunction and Oxidative Stress in Metabolic Disorders—A Step towards Mitochondria Based Therapeutic Strategies. Biochim. Biophys. Acta Mol. Basis Dis..

[2] Berra C.M., Menck C.F.M., Di Mascio P. (2006). Estresse Oxidativo, Lesões No Genoma e Processos de Sinalização No Controle Do Ciclo Celular. Quim. Nova.

[3] Mancini A., Di Segni C., Raimondo S., Olivieri G., Silvestrini A., Meucci E., Currò D. (2016). Thyroid Hormones, Oxidative Stress, and Inflammation. Mediat. Inflamm..

[4] Pugazhenthi S., Qin L., Reddy P.H. (2017). Common Neurodegenerative Pathways in Obesity, Diabetes, and Alzheimer’s Disease. Biochim. Biophys. Acta Mol. Basis Dis..

[5] Reddy P.H., Tripathi R., Troung Q., Tirumala K., Reddy T.P., Anekonda V., Shirendeb U.P., Calkins M.J., Reddy A.P., Mao P. (2012). Abnormal Mitochondrial Dynamics and Synaptic Degeneration as Early Events in Alzheimer’s Disease: Implications to Mitochondria-Targeted Antioxidant Therapeutics. Biochim. Biophys. Acta Mol. Basis Dis..

[6] Roy M., Reddy P.H., Iijima M., Sesaki H. (2015). Mitochondrial Division and Fusion in Metabolism. Curr. Opin. Cell Biol..

[7] Subramaniam S.R., Chesselet M.-F. (2013). Mitochondrial Dysfunction and Oxidative Stress in Parkinson’s Disease. Prog. Neurobiol..

[8] Guan R., Ma N., Liu G., Wu Q., Su S., Wang J., Geng Y. (2023). Ethanol Extract of Propolis Regulates Type 2 Diabetes in Mice via Metabolism and Gut Microbiota. J. Ethnopharmacol..

[9] Rodrigues G.A., Souza W.C., Godinho M.G.C., Ferreira H.D., Vila G.M. (2015). Determinação de parâmetros farmacognósticos para as folhas de *Erythroxylum suberosum* A.St.-Hil. (Erythroxylaceae) coletadas no Município de Goiânia, GO. Rev. Bras. Plantas Med..

[10] Coriolano de Oliveira E., Alves Soares Cruz R., De Mello Amorim N., Guerra Santos M., Carlos Simas Pereira Junior L., Flores Sanchez E.O., Pinho Fernandes C., Garrett R., Machado Rocha L., Lopes Fuly A. (2016). Protective Effect of the Plant Extracts of *Erythroxylum* Sp. against Toxic Effects Induced by the Venom of Lachesis Muta Snake. Molecules.

[11] Restrepo D.A., Saenz E., Jara-Muñoz O.A., Calixto-Botía I.F., Rodríguez-Suárez S., Zuleta P., Chavez B.G., Sanchez J.A., D’Auria J.C. (2019). *Erythroxylum* in Focus: An Interdisciplinary Review of an Overlooked Genus. Molecules.

[12] Lv Y., Tian T., Wang Y.-J., Huang J.-P., Huang S.-X. (2022). Advances in Chemistry and Bioactivity of the Genus *Erythroxylum*. Nat. Prod. Bioprospect..

[13] Oliveira S.L., da Silva M.S., Tavares J.F., Sena-Filho J.G., Lucena H.F.S., Romero M.A.V., Barbosa-Filho J.M. (2010). Tropane Alkaloids from *Erythroxylum* Genus: Distribution and Compilation of ^13^C-NMR Spectral Data. Chem. Biodivers..

[14] Zanolari B., Guilet D., Marston A., Queiroz E.F., Paulo M.d.Q., Hostettmann K. (2003). Tropane Alkaloids from the Bark of *Erythroxylum vacciniifolium*. J. Nat. Prod..

[15] Barros I.M.d.C., Leite B.H.M., Leite C.F.M., Fagg C.W., Gomes S.M., Resck I.S., Fonseca-Bazzo Y.M., Magalhães P.O., Silveira D. (2017). Chemical Composition and Antioxidant Activity of Extracts from *Erythroxylum suberosum* A.St. Hil.Leaves. J. Appl. Pharm. Sci..

[16] Macedo T.B.C., Elias S.T., Torres H.M., Yamamoto-Silva F.P., Silveira D., Magalhães P.O., Lofrano-Porto A., Guerra E.N.S., Silva M.A.G. (2016). Cytotoxic Effect of *Erythroxylum suberosum* Combined with Radiotherapy in Head and Neck Cancer Cell Lines. Braz. Dent. J..

[17] de Oliveira F.d.F.S., Aguiar P.N.C., Ribeiro G.d.J.G., de Amorim M.L.L., Guimarães P.S.S., Mendonça Filho C.V., Sivieri R.R.G., Brandão M.d.G.L., dos Santos W.T.P., Grael C.F.F. (2015). Antioxidant Activity and Phytochemical Screening of Extracts of *Erythroxylum suberosum* A.St.-Hil (Erythroxylaceae). Res. J. Phytochem..

[18] Aguiar J.S., Araújo R.O., Do Desterro Rodrigues M., Sena K.X.F.R., Batista A.M., Guerra M.M.P., Oliveira S.L., Tavares J.F., Silva M.S., Nascimento S.C. (2012). Antimicrobial, Antiproliferative and Proapoptotic Activities of Extract, Fractions and Isolated Compounds from the Stem of *Erythroxylum caatingae* Plowman. Int. J. Mol. Sci..

[19] Silva G.L., Cui B., Chávez D., You M., Chai H.-B., Rasoanaivo P., Lynn S.M., O’Neill M.J., Lewis J.A., Besterman J.M. (2001). Modulation of the Multidrug-Resistance Phenotype by New Tropane Alkaloid Aromatic Esters from *Erythroxylum pervillei*. J. Nat. Prod..

[20] Mahomoodally M.F., Gurib-Fakim A., Subratty A.H. (2005). Antimicrobial Activities and Phytochemical Profiles of Endemic Medicinal Plants of Mauritius. Pharm. Biol..

[21] Barreiros A.L.B., Barreiros M.L., David J.M., David J.P., Queiroz L.P. (2003). de Atividade Antioxidante de Substâncias Presentes Em Dioclea Violacea e *Erythroxylum nummularia*. Rev. Bras. Farmacogn..

[22] Barreiros M.L., David J.P., David J.M., Xavier Lopes L.M., de Sá M.S., Costa J.F.O., Almeida M.Z., de Queiróz L.P., Sant’Ana A.E.G. (2007). Ryanodane Diterpenes from Two *Erythroxylum* Species. Phytochemistry.

[23] Barreiros M.L., David J.M., de Queiroz L.P., David J.P. (2005). Flavonoids and Triterpenes from Leaves of *Erythroxylum nummularia*. Biochem. Syst. Ecol..

[24] Panzella L. (2023). Polyphenols and Their Impact on Human Health. Int. J. Mol. Sci..

[25] Martiniakova M., Babikova M., Mondockova V., Blahova J., Kovacova V., Omelka R. (2022). The Role of Macronutrients, Micronutrients and Flavonoid Polyphenols in the Prevention and Treatment of Osteoporosis. Nutrients.

[26] Wianowska D., Olszowy-Tomczyk M. (2023). A Concise Profile of Gallic Acid—From Its Natural Sources through Biological Properties and Chemical Methods of Determination. Molecules.

[27] Naoi M., Wu Y., Shamoto-Nagai M., Maruyama W. (2019). Mitochondria in Neuroprotection by Phytochemicals: Bioactive Polyphenols Modulate Mitochondrial Apoptosis System, Function and Structure. Int. J. Mol. Sci..

[28] Kumar S., Pandey A.K. (2013). Chemistry and Biological Activities of Flavonoids: An Overview. Sci. World J..

[29] Hussen E.M., Endalew S.A. (2023). In Vitro Antioxidant and Free-Radical Scavenging Activities of Polar Leaf Extracts of Vernonia Amygdalina. BMC Complement. Med. Ther..

[30] Wutsqa Y.U., Suratman S., Sari S.L.A. (2021). Detection of Terpenoids and Steroids in Lindsaea Obtusa with Thin Layer Chromatography. Asian J. Nat. Prod. Biochem..

[31] Jain D., Meena M., Janmeda P., Seth C., Arora J. (2024). Analysis of quantitative phytochemical content and Antioxidant Activity of leaf, stem, and bark of *Gymnosporia senegalensis* (Lam.) Loes. Plants.

[32] Uddin M.J., Debnath B., Patari P., Nag S.K., Sil S.K., Manna K. (2021). Ethnomedicinal Survey and Determination of Total Alkaloids and Phenolics in Selected Edible Plants of Tripura, India. J. Med. Plants Stud..

[33] Shamsa F., Monsef H., Ghamooshi R., Verdian-rizi M. (2008). Spectrophotometric Determination of Total Alkaloids in Some Iranian Medicinal Plants. Thai J. Pharm. Sci..

[34] Ghasemzadeh A., Jaafar H.Z.E., Rahmat A., Ashkani S. (2015). Secondary Metabolites Constituents and Antioxidant, Anticancer and Antibacterial Activities of *Etlingera elatior* (Jack) R.M.Sm Grown in Different Locations of Malaysia. BMC Complement. Altern. Med..

[35] Moreira B.O., Barbosa Filho M.R.D., de Carvalho A.L., da Silva D.G., Cruz M.P., Yatsuda R., David J.M. (2020). Application of Response Surface Methodology for Optimization of Ultrasound-Assisted Solid-Liquid Extraction of Phenolic Compounds from Cenostigma Macrophyllum. J. Chemom..

[36] Moreira B.O., Vilar V.L.S., de Almeida R.N.S., Morbeck L.L.B., Andrade B.S., Barros R.G.M., Neves B.M., de Carvalho A.L., Cruz M.P., Yatsuda R. (2022). New Dimer and Trimer of Chalcone Derivatives from Anti-Inflammatory and Antinociceptive Extracts of Schinopsis Brasiliensis Roots. J. Ethnopharmacol..

[37] Moreira B.O., De Carvalho A.L., Alves C.Q., Morbeck L.L.B., Cruz M.P., Yatsuda R., David J.P., David J.M. (2019). Evaluation of Anti-Inflammatory, Antinociceptive and Biological Activities of: Cenostigma Macrophyllum Standardized Extracts and Determination and Quantification of the Main Metabolites. RSC Adv..

[38] Brand-Williams W., Cuvelier M.E., Berset C. (1995). Use of a Free Radical Method to Evaluate Antioxidant Activity. LWT Food Sci. Technol..

[39] Jan S., Khan M.R., Rashid U., Bokhari J. (2013). Assessment of Antioxidant Potential, Total Phenolics and Flavonoids of Different Solvent Fractions of *Monotheca buxifolia* Fruit. Osong Public Health Res. Perspect..

[40] Shah N.A., Khan M.R., Ahmad B., Noureen F., Rashid U., Khan R.A. (2013). Investigation on Flavonoid Composition and Anti Free Radical Potential of Sida Cordata. BMC Complement. Altern. Med..

[41] Campos G.B., Souza S.G., Lob O.T.N., Da Silva D.C.C., Sousa D.S., Oliveira P.S., Santos V.M., Amorim A.T., Farias S.V.T., Cruz M.P. (2012). Isolation, Molecular Characteristics and Disinfection of Methicillin-Resistant *Staphylococcus aureus* from ICU Units in Brazil. New Microbiol..

[42] Yatsuda R., Rosalen P.L., Cury J.A., Murata R.M., Rehder V.L.G., Melo L.V., Koo H. (2005). Effects of *Mikania* Genus Plants on Growth and Cell Adherence of Mutans Streptococci. J. Ethnopharmacol..

[43] Malfa G.A., Pappalardo F., Miceli N., Taviano M.F., Ronsisvalle S., Tomasello B., Bianchi S., Davì F., Spadaro V., Acquaviva R. (2023). Chemical, Antioxidant and Biological Studies of *Brassica incana* Subsp. *Raimondoi* (Brassicaceae) Leaf Extract. Molecules.

[44] Simeonova R., Zheleva D., Valkova I., Stavrakov G., Philipova I., Atanasova M., Doytchinova I. (2021). A Novel Galantamine-Curcumin Hybrid as a Potential Multi-Target Agent against Neurodegenerative Disorders. Molecules.

[45] Zhang Y., Meng X., Shen Y., Xie J., Yu X., Wang Q., Wang L. (2021). The Reliability and Validity of the Brief ICF Core Set in Patients with Chronic Obstructive Pulmonary Disease. Int. J. Chron. Obstruct. Pulmon. Dis..

[46] Kuhns B.D., McCarroll T.R., Quesada-Jimenez R., Kahana-Rojkind A.H., Sikligar D., Cohen M.F., Domb B.G. (2025). Preoperative Anteroposterior and Lateral Assessment of Sagittal Spinopelvic Parameters Show High Positional Correlation and Measurement Reliability Preceding Both Hip Preservation and Reconstruction Surgery. Arthrosc. Sport. Med. Rehabil..

[47] David J.P., Silva E.F.D., Moura D.L.D., Guedes M.L.D.S., Assunção R.D.J., David J.M. (2001). Lignanas e triterpenos do extrato citotóxico de eriope blanchetii. Quim. Nova.

[48] Hamimed S., Boulebda N., Laouer H., Belkhiri A. (2025). Bioactivity-Guided Isolation of Alkamides from a Cytotoxic Fraction of the Ethyl Acetate Extract of *Anacyclus pyrethrum* (L.) DC. Roots. Curr. Issues Pharm. Med. Sci..

[49] Mamoona, Nosheen S., Riaz S., Shah S.I., Shahid S. (2025). Optimizing Extraction Methods: The Role of Solvent Polarity in Enhancing Phenolic Content and Antioxidant Activity in Biowaste. Biomass Convers. Biorefin..

[50] Liu L., Suo T. (2025). Review, Challenges, and Prospects of the Process of Alkaloid Extraction from Plants. Chem. Eng. Process. Process Intensif..

[51] Cheng Y., Kang Y., Kim W. (2025). Solvent Fractionation of Polygonum Cuspidatum Sieb. et Zucc. for Antioxidant, Biological Activity, and Chromatographic Characterization. Int. J. Mol. Sci..

[52] Sultana T., Ahmed M., Akhtar N., Okla M.K., Al-Hashimi A., Al-Qahtani W.H., Abdelgawad H., Ihsan-ul-Haq (2022). Polarity Directed Appraisal of Pharmacological Potential and HPLC-DAD Based Phytochemical Profiling of *Polygonum glabrum* Willd. Molecules.

[53] Shanmugam N., Adam S.K., Rahman S.A., Moklas M.A.M. (2018). Phytochemical Screening and Antioxidant Activities of *Erythroxylum cuneatum* Leaf Extracts. Nat. Prod. J..

[54] Adam S.K., Shanmugam N., Mohamad S., Rahman S.A., Moklas M.A.M. (2023). In Vitro Antioxidant and Anti-Inflammatory Effects of *Erythroxylum cuneatum* Leaf Extract on Oxidized Low-Density Lipoprotein-Stimulated Human Aortic Endothelial Cells. Pharmacogn. Mag..

[55] Li L.S., Chiroma S.M., Hashim T., Adam S.K., Mohd Moklas M.A., Yusuf Z., Rahman S.A. (2020). Antioxidant and Anti-Inflammatory Properties of *Erythroxylum cuneatum* Alkaloid Leaf Extract. Heliyon.

[56] Marentes-Culma R., Orduz-Díaz L.L., Lozano-Garzón K., Carrillo M.P. (2025). From Tradition to Science: Chemical, Nutritional, and Cytotoxic Characterization of *Erythroxylum coca* from Indigenous Colombian Communities. ACS Omega.

[57] Neergheen V.S., Bahorun T., Jen L.-S., Aruoma O.I. (2007). Bioefficacy of Mauritian Endemic Medicinal Plants: Assessment of Their Phenolic Contents and Antioxidant Potential. Pharm. Biol..

[58] Aghoutane B., Talbi H., Naama A., Monfalouti H.E., Kartah B.E. (2023). Effect of Extraction Solvent on Total Phenol Content, Total Flavonoid Content, and Antioxidant Activity of *Euphorbia resinifiera* O. Berg. Trop. J. Nat. Prod. Res..

[59] Ogundele A.V., Das A.M., Paz C. (2025). Gallic Acid from Elaeocarpus Floribundus Stem Bark: A Potent Natural Antioxidant with Enzymatic and Pharmacokinetic Validation. Antioxidants.

[60] Bouakline H., Bouknana S., Merzouki M., Ziani I., Challioui A., Bnouham M., Tahani A., ELBachiri A. (2024). The Phenolic Content of Pistacia Lentiscus Leaf Extract and Its Antioxidant and Antidiabetic Properties. Sci. World J..

[61] Yu J., Xiao X., Chen B., Deng Z., Chen X., Fan Y., Li H. (2024). Synergistic and Antagonistic Activity of Selected Dietary Phytochemicals against Oxidative Stress-Induced Injury in Cardiac H9c2 Cells via the Nrf2 Signaling Pathway. Foods.

[62] Parveen Z., Zaidi S., Bajguz A., Arif Y., Hayat S. (2025). Comprehensive Insights into Flavonoids: Biosynthesis, Stress Modulation, and Plant Growth Regulation. J. Plant Growth Regul..

[63] Chen J., Yang J., Ma L., Li J., Shahzad N., Kim C.K. (2020). Structure-Antioxidant Activity Relationship of Methoxy, Phenolic Hydroxyl, and Carboxylic Acid Groups of Phenolic Acids. Sci. Rep..

[64] Bencheikh N., Bouhrim M., Merrouni I.A., Boutahiri S., Kharchoufa L., Addi M., Tungmunnithum D., Hano C., Eto B., Legssyer A. (2021). Antihyperlipidemic and Antioxidant Activities of Flavonoid-Rich Extract of *Ziziphus lotus* (L.) Lam. Fruits. Appl. Sci..

[65] Nzekwe S., Morakinyo A., Ntwasa M., Lebelo S., Oguntibeju O., Oyedapo O., Ayeleso A. (2024). Flavonoid-Rich Fraction of Monodora Tenuifolia Benth Seeds Improves Antioxidant Status in Male Wistar Rats with Streptozotocin-Induced Diabetes Mellitus. Phytomed. Plus.

[66] Kiokias S., Oreopoulou V. (2022). Review on the Antioxidant Activity of Phenolics in o/w Emulsions along with the Impact of a Few Important Factors on Their Interfacial Behaviour. Colloids Interfaces.

[67] Chen Z., Świsłocka R., Choińska R., Marszałek K., Dąbrowska A., Lewandowski W., Lewandowska H. (2024). Exploring the Correlation Between the Molecular Structure and Biological Activities of Metal–Phenolic Compound Complexes: Research and Description of the Role of Metal Ions in Improving the Antioxidant Activities of Phenolic Compounds. Int. J. Mol. Sci..

[68] Kiss A., Papp V.A., Pál A., Prokisch J., Mirani S., Toth B.E., Alshaal T. (2025). Comparative Study on Antioxidant Capacity of Diverse Food Matrices: Applicability, Suitability and Inter-Correlation of Multiple Assays to Assess Polyphenol and Antioxidant Status. Antioxidants.

[69] Rush J.S., Zamakhaeva S., Murner N.R., Deng P., Morris A.J., Kenner C.W., Black I., Heiss C., Azadi P., Korotkov K.V. (2025). Structure and Mechanism of Biosynthesis of *Streptococcus mutans* Cell Wall Polysaccharide. Nat. Commun..

[70] Loyola D., Mendoza R., Chiong L., Rueda M., Alvítez-Temoche D., Gallo W., Mayta-Tovalino F. (2020). Ethanol Extract of *Schinus molle* L. (Molle) and *Erythroxylum coca* Lam (Coca): Antibacterial Properties at Different Concentrations against: Streptococcus Mutans: An: In Vitro: Study. J. Int. Soc. Prev. Community Dent..

[71] Cushnie T.P.T., Lamb A.J. (2005). Antimicrobial Activity of Flavonoids. Int. J. Antimicrob. Agents.

[72] Daglia M. (2012). Polyphenols as Antimicrobial Agents. Curr. Opin. Biotechnol..

[73] Villanueva X., Zhen L., Ares J.N., Vackier T., Lange H., Crestini C., Steenackers H.P. (2023). Effect of Chemical Modifications of Tannins on Their Antimicrobial and Antibiofilm Effect against Gram-Negative and Gram-Positive Bacteria. Front. Microbiol..

[74] Hurtado-Díaz I., Ramírez-Cisneros M.Á., Alvarez L., Sánchez-Carranza J.N., Columba-Palomares M.C., Silva-Guzmán J.A., Cruz-Sosa F., Bernabé-Antonio A. (2024). Metabolites Profile of Extracts and Fractions of *Erythroxylum mexicanum* Kunth by UHPLC-QTOF-MS/MS and Its Antibacterial, Cytotoxic and Nitric Oxide Inhibitory Activities. Chem. Biodivers..

[75] Ntungwe N E., Domínguez-Martín E.M., Roberto A., Tavares J., Isca V., Pereira P., Cebola M.J., Rijo P. (2020). *Artemia* Species: An Important Tool to Screen General Toxicity Samples. Curr. Pharm. Des..

[76] Spinelli R., Rietmann Á., Sanchis I., Goicoechea H., Siano Á. (2024). Toxicity Evaluation of Anti-Cholinesterasic Amphibian Extracts by MTT and an Optimized *Artemia salina* Test. Chem. Biodivers..

[77] Rahamouz-Haghighi S., Bagheri K., Sharafi A., Tavakolizadeh M., Mohsen-Pour N. (2022). Phytochemical Screening and Cytotoxicity Assessment of *Plantago lanceolata* L. Root Extracts on Colorectal Cancer Cell Lines and Brine Shrimp Larvae and Determination of the Median Lethal Dose in Mice. S. Afr. J. Bot..

[78] Rajabi S., Ramazani A., Hamidi M., Naji T. (2015). *Artemia salina* as a Model Organism in Toxicity Assessment of Nanoparticles. DARU J. Pharm. Sci..

[79] Elias S.T., Macedo C.C.S., Simeoni L.A., Silveira D., Magalhães P.O., Lofrano-Porto A., Coletta R.D., Neves F.A.R., Guerra E.N.S. (2016). Cytotoxic Effect of *Erythroxylum daphnites* Extract Is Associated with G1 Cell Cycle Arrest and Apoptosis in Oral Squamous Cell Carcinoma. Cell Cycle.

[80] de Jesus Menezes-Filho N., Souza C.D.S., Costa T.C.S., Da Silva V.D.A., de Oliveira Ribeiro C.S., Barreiros M.L., Costa J.F.O., David J.M., David J.P., Costa S.L. (2014). Cytotoxicity of the Diterpene 14-O-Methyl-Ryanodanol from *Erythroxylum passerinum* in an Astrocytic Cells Model. Nat. Prod. Commun..

[81] da Silva L.J.C., Alves L.A., Silva V.R., Santos L.S., Bezerra D.P., Soares M.B.P., Doriguetto A.C., Barbosa L.C.A., do Nascimento J.C., Macedo G.E.L. (2021). A New Tropane Alkaloid and Other Metabolites from *Erythroxylum macrocalyx* (Erythroxylaceae) and Their Antiproliferative Activities. Phytochem. Lett..

[82] Brito L.S.d.O., Pinto F.d.C.L., de Filho M.O.M., Rocha D.D., Mendoza M.F.M., Ayala A.P., Bezerra B.P., Loiola M.I.B., Canuto K.M., Silveira E.R. (2020). Tropane Alkaloids from the Stem Bark of *Erythroxylum bezerrae*. Phytochemistry.

[83] Olofinsan K., Abrahamse H., George B.P. (2023). Therapeutic Role of Alkaloids and Alkaloid Derivatives in Cancer Management. Molecules.

[84] Pyo Y., Kwon K.H., Jung Y.J. (2024). Anticancer Potential of Flavonoids: Their Role in Cancer Prevention and Health Benefits. Foods.

[85] Kamran S., Sinniah A., Abdulghani M.A.M., Alshawsh M.A. (2022). Therapeutic Potential of Certain Terpenoids as Anticancer Agents: A Scoping Review. Cancers.

[86] Ju J., Li Z., Liu J., Peng X., Gao F. (2025). Biased Opioid Receptor Agonists: Balancing Analgesic Efficacy and Side-Effect Profiles. Int. J. Mol. Sci..

[87] Quiñonez-Bastidas G.N., Pineda-Farias J.B., Flores-Murrieta F.J., Rodríguez-Silverio J., Reyes-García J.G., Godínez-Chaparro B., Granados-Soto V., Rocha-González H.I. (2018). Antinociceptive Effect of (−)-Epicatechin in Inflammatory and Neuropathic Pain in Rats. Behav. Pharmacol..

[88] Takeda M., Sashide Y., Toyota R., Ito H. (2024). The Phytochemical, Quercetin, Attenuates Nociceptive and Pathological Pain: Neurophysiological Mechanisms and Therapeutic Potential. Molecules.

[89] Itou H., Toyota R., Takeda M. (2022). Phytochemical Quercetin Alleviates Hyperexcitability of Trigeminal Nociceptive Neurons Associated with Inflammatory Hyperalgesia Comparable to NSAIDs. Mol. Pain.

[90] Lim E.Y., Lee C., Kim Y.T. (2022). The Antinociceptive Potential of *Camellia japonica* Leaf Extract, (−)-Epicatechin, and Rutin against Chronic Constriction Injury-Induced Neuropathic Pain in Rats. Antioxidants.

[91] Miaffo D., Kamgue O.G., Kolefer K., Dadaya E., Mahamad T.A., Maidadi B., Kamanyi A. (2025). Acute and Subacute Toxicity of the Aqueous Extract of Cissus Polyantha Glig and Bradt (Vitaceae) on Biochemical and Hematological Parameters in Wistar Rats. BMC Complement. Med. Ther..

[92] Ali S.S., Al-Tohamy R., Al-Zahrani M., Badr A., Sun J. (2025). Essential Oils and Plant-Derived Bioactive Compounds: A Comprehensive Review of Their Therapeutic Potential, Mechanisms of Action, and Advances in Extraction Technologies. Phytochem. Rev..

[93] May J.E., Marques M.B., Reddy V.V.B., Gangaraju R. (2019). Three Neglected Numbers in the CBC: The RDW, MPV, and NRBC Count. Cleve. Clin. J. Med..

[94] Hadidi M., Liñán-Atero R., Tarahi M., Christodoulou M.C., Aghababaei F. (2024). The Potential Health Benefits of Gallic Acid: Therapeutic and Food Applications. Antioxidants.

[95] Khan A.K., Rashid R., Fatima N., Mahmood S., Mir S., Khan S., Jabeen N., Murtaza G. (2015). Pharmacological Activities of Protocatechuic Acid. ACTA Pol. Pharm..

[96] Li S.S., Fan Y.H., Shu C.Y., Zhou Y.R., Shu J. (2024). Methyl 3,4-Dihydroxybenzoate Alleviates Oxidative Damage in Granulosa Cells by Activating Nrf2 Antioxidant Pathway. J. Ovarian Res..

[97] Andrés C.M., Pérez de la Lastra J.M., Juan C.A., Plou F.J., Pérez-Lebeña E. (2023). Polyphenols as Antioxidant/Pro-Oxidant Compounds and Donors of Reducing Species: Relationship with Human Antioxidant Metabolism. Processes.

[98] Kiokias S., Proestos C., Oreopoulou V. (2020). Phenolic Acids of Plant Origin—A Review on Their Antioxidant Activity In Vitro (O/W Emulsion Systems) Along with Their in Vivo Health Biochemical Properties. Foods.

[99] Mączka W., Duda-Madej A., Grabarczyk M., Wińska K. (2022). Natural Compounds in the Battle against Microorganisms—Linalool. Molecules.

[100] Panchal P., Miller A.J., Giri J. (2021). Organic Acids: Versatile Stress-Response Roles in Plants. J. Exp. Bot..

