# Supplementary material for: Phytochemical Profile and Bioactive Potential of Erythroxylum nummularium Peyr: Evaluation of Antioxidant, Antimicrobial, and Antinociceptive Properties
Source: Antioxidants (Basel). 2026 Aug 18;15(8):1027. doi: 10.3390/antiox15081027 (PMC13509508; doi:10.3390/antiox15081027)
Supplement: Supplementary file 1 [file antioxidants-15-01027-s001.zip › antioxidants-4287868-supplementary.pdf]

Supplementary Materials

Phytochemical Profile and Bioactive Potential of *Erythroxylum nummularium* Peyr: Evaluation of Antioxidant, Antimicrobial, and Antinociceptive Properties

Nilma Santos da Silva [1], Ícaro Tuyá Caires Amorim [1], Flávio Mendes de Souza [1], Brenda Oliveira Lima [1], Talita Costa dos Santos [1], Djalma Menezes de Oliveira [2], Lucas Miranda Marques [1], Mariluze Peixoto Cruz [1], Anaildes Lago de Carvalho [3], Regiane Yatsuda [1] and Bruno Oliveira Moreira [1,*]

[1] Multidisciplinary Institute in Health, Federal University of Bahia, Center for Natural Sciences and Biodiversity, Vitória da Conquista 45029-094, BA, Brazil; lucasm@ufba.br (L.M.M.); mariluze@ufba.br (M.P.C.); yatsuda@ufba.br (R.Y.)
[2] Department of Science and Technology, State University of Southwest Bahia, Jequié 45208-091, BA, Brazil
[3] Department of Natural Sciences, State University of Southwest Bahia, Vitória da Conquista 45031-300, BA, Brazil; anaildes.carvalho@uesb.edu.br
* Correspondence: bmoreira@ufba.br

Table S1. Chemical constituents identified by GC–MS in derivatized extracts of *E. nummularium*.

Fractions	Identified compounds	%Área	M+	Formula	Rt
DLEN	Glycerol	13.35	308	$C_3H_8O_3$	14.055
	Palmitic acid	8.51	328	$C_{16}H_{32}O_2$	35.931
	Linalool oxide	6.35	170	$C_{10}H_{18}O_2$	17.981
	3,4-dihydroxybenzoic acid	1.25	370	$C_7H_6O_4$	30.703
	Methyl 3,4-dihydroxybenzoate	1.98	168	$C_8H_8O_4$	28.036
	Glycerol-3-phosphate	0.90	460	$C_{15}H_{41}O_6PSi_4$	29.665
	Stearic acid	0.50	356	$C_{18}H_{34}O_2$	40.309
	Azelaic acid	0.94	332	$C_9H_{16}O_4$	30.022
	2,3-Dihydroxypropyl hexadecanoate	7.18	330	$C_{19}H_{38}O_4$	47.356
HLEN	Palmitic acid	23.67	328	$C_{16}H_{32}O_2$	35.931
	Neophytadiene	0.51	278	$C_{20}H_{38}$	30.819
	(6R)-6,10,14-trimethylpentadecan-2-one	0.22	268	$C_{18}H_{36}O$	31.045
	Tetradecanoic acid	0.62	300	$C_{14}H_{28}O_2$	31.166

	Methyl hexadecanoate	16.58	270	$C_{17}H_{34}O_2$	33.117
	Methyl heptadecanoate	0.61	284	$C_{18}H_{36}O_2$	35.455
	Methyl linoleate	2.64	294	$C_{19}H_{34}O_2$	37.022
	Methyl oleate	6.84	296	$C_{19}H_{36}O_2$	37.177
	Elaidic acid	8.52	354	$C_{18}H_{34}O_2$	39.784
	Methyl octadecanoate	6.16	298	$C_{19}H_{38}O_2$	37.763
	Methyl oleate	6,84	296	$C_{18}H_{31}ClO$	37.177
	Heptadecanoic acid	0.48	270	$C_{17}H_{34}O_2$	35.472
	Butyl hexadecanoate	0.43	312	$C_{20}H_{40}O_2$	39.035
	Linoleic acid	3.72	352	$C_{18}H_{32}O_2$	39.627
	Stearic acid	3.84	356	$C_{18}H_{36}O_2$	40.309
	Docosane	0.45	296	$C_{21}H_{44}$	43.401
	Tetracosane	0.73	338	$C_{24}H_{50}$	45.363
	Tetratriacontane	0.73	478	$C_{34}H_{70}$	47.241
	1-Monopalmitin trimethylsilyl ether	0.64	474	$C_{25}H_{54}O_4Si_2$	47.352
	β-sitosterol	4.18	414	$C_{29}H_{50}O$	60.531
	β-amyrin	3.12	498	$C_{30}H_{50}O$	60.724
	Lupeol acetate	1.81	468	$C_{32}H_{52}O_2$	61.846
DBEN	Glycerol	5.31	308	$C_3H_8O_3$	14.055
	Methyl 3,4-dihydroxybenzoate	2.75	168	$C_8H_8O_4$	28.036
	3-Hydroxypropanoic acid	0.43	90	$C_3H_6O_3$	9.646
	Propane-1,3-diol	0.98	76	$C_3H_8O_2$	14.112
	Succinic acid	0.99	118	$C_4H_6O_4$	15.681
	Oxalic acid	1.47	90	$C_2H_2O_4$	17.726
	Malic acid	2.63	134	$C_4H_6O_5$	21.63
	4-Hydroxy-3-methoxybenzoic acid	0.56	168	$C_8H_8O_4$	29.22
	3,4-dihydroxybenzoic acid	1.97	154	$C_7H_6O_4$	30.719
	Palmitic acid	0.41	328	$C_{16}H_{32}O_2$	35.931
	Glyceric acid	1.64	106	$C_3H_6O_4$	16.488
HBEN	Methyl hexadecanoate	16.59	270	$C_{17}H_{34}O_2$	33.117
	Methyl heptadecanoate	0.99	284	$C_{18}H_{36}O_2$	35.455
	Stearic acid	1.65	356	$C_{18}H_{36}O_2$	40.309
	Methyl linoleate	5.88	294	$C_{19}H_{34}O_2$	37.022
	Methyl stearate	2.51	298	$C_{19}H_{38}O_2$	37.758
	Heptadecanoic acid	0.33	270	$C_{17}H_{34}O_2$	35.472
	Linoleic acid	2.37	352	$C_{18}H_{32}O_2$	39.627
	Elaidic acid	8.36	354	$C_{18}H_{34}O_2$	39.784
	Stearic acid	1.65	356	$C_{18}H_{36}O_2$	40.309
	3-Hydroxypropyl palmitate	0.40	314	$C_{19}H_{38}O_3$	45.368

	Methyl docosanoate	0.33	354	$C_{23}H_{46}O_2$	46.035
	2,3-Dihydroxypropyl hexadecanoate	1.16	330	$C_{19}H_{38}O_4$	47.356
	Palmitic acid	9.99	328	$C_{16}H_{32}O_2$	35.931
	β-sitosterol	6.22	414	$C_{29}H_{50}O$	60.531
	Methyl tetracosanoate	0.46	382	$C_{25}H_{50}O_2$	49.719
	Glyceryl monooleate	1.08	356	$C_{21}H_{40}O_4$	50.39
	Campesterol	1.33	400	$C_{28}H_{48}O$	58.665
	Stigmasterol	1.67	484	$C_{32}H_{56}OSi$	59.286
	Germanicol	3.69	426	$C_{30}H_{50}O$	60.259
	β-amyrin	1.12	498	$C_{30}H_{50}O$	60.724
	Lupenone	0.65	424	$C_{30}H_{48}O$	61.021
	Lupeol acetate	11.41	468	$C_{32}H_{52}O_2$	61.846
DSBEN	Butane-1,3-diol	2.5	90	$C_4H_{10}O_2$	14.131
	Glycerol	1.93	308	$C_3H_8O_3$	14.055
	Butane-1,3-diol, 1-methylene-3-methyl	7.81	260	$C_{12}H_{28}O_2Si_2$	18.004
	Malic acid	1.06	134	$C_4H_6O_5$	21.63
	Butane-1,2,3-Tetrol	0.42	122	$C_4H_{10}O_4$	22.405
	p-Hidroxybenzoic acid	0.56	138	$C_7H_6O_3$	25.365
	Methyl 3,4-dihydroxybenzoate	7.49	168	$C_8H_8O_4$	28.036
	4-Hydroxy-3-methoxybenzoic acid	0.95	168	$C_8H_8O_4$	29.22
	3,4-dihydroxybenzoic acid	1.61	154	$C_7H_6O_4$	30.719
	Gallic acid	6.62	170	$C_7H_6O_5$	34.327
	2,3-Dihydroxypropyl hexadecanoate	0.56	330	$C_{19}H_{38}O_4$	47.356
	Glyceryl monooleate	1.37	356	$C_{21}H_{40}O_4$	50.39
	Methyl hexadecanoate	16.14	270	$C_{17}H_{34}O_2$	33.117
	Heptadecanoic acid	0.95	270	$C_{17}H_{34}O_2$	35.472
	Palmitic acid	7.60	328	$C_{16}H_{32}O_2$	35.931
	Methyl octadecanoate	19.93	298	$C_{19}H_{38}O_2$	37.763
	Methyl oleate	3.12	296	$C_{18}H_{31}ClO$	37.177
	Ácid (9Z)-Octadeca-9-enoico	9.27	354	$C_{21}H_{42}O_2Si$	39.75
	Ácid (11Z)-Octadeca-11-enoico	1.05	354	$C_{21}H_{42}O_2Si$	39.876
	Methyl docosanoate	1.32	354	$C_{23}H_{46}O_2$	46.028
	Methyl tetracosanoate	2.23	382	$C_{25}H_{50}O_2$	49.719
	Campesterol	1.25	400	$C_{28}H_{48}O$	58.665
	Stigmasterol	1.98	484	$C_{29}H_{48}O$	59.286

	β-amyrin	0.95	498	$C_{30}H_{50}O$	60.724
	β-sitosterol	6.21	414	$C_{29}H_{50}O$	60.531
	Gorgosterol	1.43	426	$C_{30}H_{50}O$	60.924
	Lupenone	0.98	424	$C_{30}H_{48}O$	61.021
	Lupeol acetate	9.14	468	$C_{32}H_{52}O_2$	61.846
HSBEN	7,10-Hexadecadienoic acid	0.92	266	$C_{16}H_{28}O_2$	32.003
	Methyl hexadecanoate or Methyl palmitate	16.14	270	$C_{17}H_{34}O_2$	33.117
	Heptadecanoic acid	0.95	270	$C_{17}H_{34}O_2$	35.472
	Hexadecanoic acid, trimethylsilyl ester	7.60	328	$C_{19}H_{40}O_2Si$	35.095
	9,12-Octadecadienoic acid (Z,Z)-, methyl ester palmitic acid	6.56	294	$C_{19}H_{34}O_2$	37.038
	Methyl (E)-Octadec-11-enoate	19.93	296	$C_{19}H_{36}O_2$	37.213
	9-Octadecenoid acid, methyl ester	3.12	296	$C_{19}H_{36}O_2$	37.297
	Methyl octadecanoate	3.06	298	$C_{19}H_{38}O_2$	37.763
	9,12-Octadecadienoic acid (Z,Z)-, trimetylsilyl ester	2.13	352	$C_{21}H_{40}O_2Si$	39.62
	Trimethylsilyl (9E)-9-Octadecenoate	9.27	354	$C_{21}H_{42}O_2Si$	39.75
	11-trans-Octadecenoic acid, trimethylsilyl	1.05	354	$C_{21}H_{42}O_2Si$	39.876
	Methyl docosanoate	1.32	354	$C_{23}H_{46}O_2$	46.028
	2,3-Bis (trimethylsilyloxy) propyl hexadecanoate	0.79	474	$C_{25}H_{54}O_4Si_2$	47.357
	Methyl tetracosanoate	2.23	382	$C_{25}H_{50}O_2$	49.719
	1-Monooleoylglycerol trimethylsilyl ether	1.27	500	$C_{27}H_{56}O_4Si_2$	50.391
	Campesterol Tms	1.25	472	$C_{31}H_{56}OSi$	58.665
	Stigmasterol Tms	1.98	484	$C_{32}H_{56}OSi$	59.286
	β-Sitosterol	6.21	486	$C_{29}H_{50}O$	60.535
	β-amyrin Tms	0.95	498	$C_{33}H_{58}OSi$	60.007
	Lupenone	0.98	424	$C_{30}H_{48}O$	61.021
	Lupeol acetate	9.14	468	$C_{32}H_{52}O_2$	61.846
DSEN	4-Hydroxy-3-methoxybenzoic acid	0.62	168	$C_8H_8O_4$	29.245
	Methyl palmitate	5.42	270	$C_{17}H_{34}O_2$	33.117
	Palmitic acid	5.42	328	$C_{16}H_{32}O_2$	35.931
	Methyl linoleate	3.02	294	$C_{19}H_{34}O_2$	37.022
	(11E)-Octadec-11- Methyl enoate	7.72	296	$C_{19}H_{36}O_2$	37.159
	Heptadecanoic acid	1.05	270	$C_{17}H_{34}O_2$	35.472

Linoleic acid	4.49	352	$C_{18}H_{32}O_2$	39.627
Elaidic acid	15.93	354	$C_{18}H_{34}O_2$	39.784
(E)-Octadec-11-enoic acid	1.53	282	$C_{18}H_{34}O_2$	39.087
Glyceryl monooleate	2.01	356	$C_{21}H_{40}O_4$	50.39
Campesterol Tms	1.70	472	$C_{31}H_{56}OSi$	58.665
β-Sitosterol	7.59	414	$C_{29}H_{50}O$	60.531
Lupeol acetate	1.79	468	$C_{32}H_{52}O_2$	61.846

Table S2. Hematological parameters after 15 days of treatment with the ELEN fraction (2000 mg·kg^{-1}). Values are expressed as mean ± standard deviation (n = 6). Reference ranges are provided for comparison. Data were obtained using an automated hematological analyzer.

Complete blood count	Control	ELEN	Reference values
WBC x 10^3/L	4.8 ± 0.62	2.9 ±1.77	2.9-15.3
LYM × 10^3/L	3.4 ± 0.56	2.8 ± 1.58	2.6–13.5
MID x 10^3/L	0.7 ± 0.04	0.2 ± 0,08	up to 0.8
GRA × 10^3/L	0.6 ± 0.03	0.37 ± 0,12	0.4–3.2
LYM %	71.6 ± 2.3	76.13 ± 6.67	63.7–90.1
MID %	15.5 ± 1.2	9.8 ± 3.77	10–20
GRA %	12.9 ± 1.6	12.13 ± 2.15	7.3–30.1
RBC	7.79 ± 0.3	7.23 ± 2.56	5.6–7.89
HGB	14.4 ± 7.8	11.6 ± 3.96	14.5–16.9
HCT %	40.4 ± 1.8	35.47 ± 12.52	36–46
MCV fl	46.9 ± 1.2	49.2 ± 0.14	47.1–47.8
MCH pg	19.5 ± 1.3	16.1 ± 0.37	16.0–23.1
MCHC g/L	32.5 ± 5.4	32.9 ± 0.70	32.4–32.8
RDW %	14.7 ± 1.1	16.93 ± 0.25	11.0–15.5
PLT × 10^3/	736 ± 159	669.33 ± 178.17	100–1610
MPV fl	6.2 ± 1.2	7.23 ± 0.31	3.8–6.2
PDW fl	7.7 ± 1.5	15.1 ± 0.24	7–23

Abbreviations: WBC, white blood cell count; LYM, lymphocytes; MID, mid-sized cells, including monocytes and other rare leukocytes; GRA, granulocytes; RBC, red blood cell count; HGB, hemoglobin; HCT, hematocrit; MCV, mean corpuscular volume; MCH, mean corpuscular hemoglobin; MCHC, mean corpuscular hemoglobin concentration; RDW, red cell distribution width; PLT, platelet count; MPV, mean platelet volume; PDW, platelet distribution width.
